# Delayed step-by-step decompression with DSF alleviates skeletal muscle crush injury by inhibiting NLRP3/CASP-1/GSDMD pathway

**DOI:** 10.1038/s41420-023-01570-3

**Published:** 2023-08-01

**Authors:** Ning Li, Xinyue Wang, Yuru Wang, Pengtao Wang, Na Sun, Jiale Chen, Lu Han, Zizheng Li, Haojun Fan, Yanhua Gong

**Affiliations:** 1 Institute of Disaster and Emergency Medicine, Medical College, Tianjin University, No. 92 Weijin Road, Nankai District, Tianjin 300072 China; 2 Tianjin Key Laboratory of Disaster Medicine Technology, Tianjin, 300072 China; 3 Department of Intensive Care Unit, Tianjin First Center Hospital, Tianjin, 300192 China

**Keywords:** Cell death, Cell death and immune response, Diseases

## Abstract

Crush injury (CI) is a common disease in earthquake and traffic accidents. It refers to long-term compression that induces ischemia and hypoxia injury of skeletal muscle rich parts, leading to rupture of muscle cells and release of contents into the blood circulation. Crush syndrome (CS) is the systemic manifestation of severe, traumatic muscle injury. CI rescue faces a dilemma. Ischemic reperfusion due to decompression is a double-edged sword for the injured. Death often occurs when the injured are glad to be rescued. Programmed cell death (PCD) predominates in muscle CI or ischemia-reperfusion injury. However, the function and mechanism of pyroptosis and apoptosis in the pathogenesis of skeletal muscle injury in CI remain elusive. Here, we identified that pyroptosis and apoptosis occur independently of each other and are regulated differently in the injured mice’s skeletal muscle of CI. While in vitro model, we found that glucose-deprived ischemic myoblast cells could occur pyroptosis. However, the cell damage degree was reduced if the oxygen was further deprived. Then, we confirmed that delayed step-by-step decompression of CI mice could significantly reduce skeletal muscle injury by substantially inhibiting NLRP3/Casp-1/GSDMD pyroptosis pathway but not altering the Casp-3/PARP apoptosis pathway. Moreover, pyroptotic inhibitor DSF therapy alone, or the combination of delayed step-by-step decompression and pyroptotic inhibitor therapy, significantly alleviated muscle injury of CI mice. The new physical stress relief and drug intervention method proposed in this study put forward new ideas and directions for rescuing patients with CI, even CS-associated acute kidney injury (CS-AKI).

## Introduction

Crush injury (CI) refers to the long-term compression injury of skeletal muscle rich parts [1], resulting in ischemia and hypoxia injury and rupture of muscle cells, and release of contents into the blood circulation and inducing crush syndrome (CS) [2, 3]. As the most abundant tissue in the human body, skeletal muscle accounts for about 40% of body mass [4]. Therefore, CI has a high incidence in natural disasters such as earthquakes [5], landslides, and man-made disasters such as wars, terrorist attacks, and explosions [6], as well as accidental injuries such as building collapses, traffic accidents [7], and severe beatings [8]. For the wounded, being lifted from the heavy objects does not mean the end of the rescue effort or even the beginning of the danger. When the compression of the muscles is suddenly relieved, blood circulation is restored, and reperfusion injury occurs. Damage-associated molecular patterns (DAMPs) flood into the bloodstream. So severe CI can lead to many complications, such as hyperkalemia, amputation, CS-associated acute kidney injury (CS-AKI), and even death [9–11]. Therefore, it severely impacts people’s life, property health, and the national economy.

The hazard of CI is related to the amount of pressure and the extrusion time. When tissue is compressed, blood flow is lost, leading to ischemia and hypoxia of skeletal muscle and ultimately inducing cell death. Therefore, the degree of muscle injury is closely associated with the duration of ischemia and hypoxia. According to Bywaters, skeletal muscle generally does not develop permanent injury within 2 h of ischemia. Some reversible cell damage occurs in the range of 2-4 h of ischemia. However, when ischemia lasting longer than 6 h will lead to irreversible tissue necrosis [12, 13]. Upon 24 h, the histological changes caused by ischemia–reperfusion (I/R) injury were the largest [14, 15]. Ischemic and hypoxia injury of the skeletal muscle remains a severe clinical problem, and currently, there is no effective therapy.

Programmed cell death (PCD) is vital in skeletal muscle injury [16, 17]. Apoptosis, the most classical type of PCD, occurs by extrinsic and intrinsic pathways. The characteristic morphological and biochemical features of apoptosis include chromatin condensation, DNA cleavage, cellular shrinkage, apoptotic body formation, and phagocytosis by macrophage [18]. Overall, orderly dismantling and eventual death triggered by apoptosis in a linear fashion. In other words, apoptosis is an immunogenically silent cell death type. Although Stratos et al. indicated that the skeletal muscle function of rat CI could restore by inhibiting caspase-mediated apoptosis [19], the underlying mechanism is unknown.

Unlike cell apoptosis, pyroptosis is a new form of PCD resulting from inflammation. Pyroptosis executioner proteins form large pores in the cell membrane, leading to cell swelling as extracellular fluid flows into the cell, and eventually, rapid cell lysis releases cell contents and DAMPs [20, 21]. Pyroptosis contains canonical and non-canonical inflammasome signaling pathways. Canonical inflammasome pathway response to DAMPs or injury signals, then assemble the cytosolic multiprotein signaling complex (for example, NLRP3) that recruits and cleave Casp-1. Both canonical and non-canonical pyroptosis could lead to DAMPs release, but only canonical inflammasome activation produces active IL-18 and IL-1β inflammatory mediators via Casp-1 [22]. Previous research indicated that pro-inflammatory pyroptosis contributes to muscle injury. Wang et al. indicated that NLRP3/GSDMD pathway medicated pyroptosis and promoted dexamethasone-induced muscle atrophy [23]. Yan et al. found that stem cell-derived exosomes prevent the pyroptosis of ischemic skeletal muscle injury [24]. However, the role and underlying mechanism of pyroptosis in skeletal muscle injury of CI is still unclear.

Traditionally, rapid diagnosis and reperfusion improve survival after CI. However, the immediate large influx of blood and oxygen during rapid decompression and skeletal muscle I/R injury triggers significant tissue destruction and sterile pro-inflammatory responses. Even a large amount of damaged skeletal muscle cell contents is released into the blood circulation, causing severe complications and compromised outcomes. Therefore, the decompression and clinical rescue of CI patients is a dilemma. However, the rescue of CI patients inevitably requires the removal of heavy objects. Therefore, we guess that slow decompression, in other words, slow increase of oxygen and blood flow in CI patients (CI/SR) plus specific drug interventions, will have a better effect. If there is a better effect, what is the specific molecular mechanism?

Firstly, in a classical mouse CI model, we found that pyroptosis and apoptosis occur in skeletal muscle. Secondly, in the in vitro glucose deprivation ischemic C2C12 myoblast cells model, we found that if the oxygen was further deprived, the cell damage degree was reduced. Next, in the mice CI model, we found that delayed step-by-step decompression can significantly alleviate skeletal muscle damage mainly by inhibiting the NLRP3/Casp-1/GSDMD pyroptosis pathway but not the Casp-3/PARP apoptosis pathways. Finally, pyroptotic inhibitor DSF treatment alone, or the synergism of delayed step-by-step decompression and DSF, are both more effective in alleviating muscle and kidney damage. Here, we identified a potential novel rescue or therapeutic strategy for patients with injury caused by CI, even CS-AKI.

## Results

### Cell pyroptosis and apoptosis both occur in the skeletal muscle of the mouse CI model

The mouse CI model was prepared using a homemade digital crush platform (Fig. S1a). Laboratory tests identify elevated serum creatine kinase (CK) as the most sensitive indicator for muscle injury evaluation. It is generally considered that a CK value exceeding 5000 U/L has severe muscle damage [25]. The serum biochemistry results showed that compared with the NC group, CK concentration increases with the extension of crush time (Fig. S1b). It has exceeded 5000 U/L at crush time for 12 h and 16 h, indicating that severe skeletal muscle damage has occurred (Fig. S1b). Meanwhile, the serum levels of myoglobin (Mb) (Fig. S1c) and lactate dehydrogenase (LDH) (Fig. S1d) in the CI group were also significantly upregulated with the extension of crush time. Among them, LDH, as an essential molecule marker of pyroptosis, increased dramatically within 0.5 h (*P* < 0.001), which prompted the possibility of cell pyroptosis in the muscle of CI. Moreover, HE staining revealed that in the NC group, the skeletal muscle had clear horizontal stripes, a flat cut surface, dense tissue, and many nuclei (Fig. S1e). In contrast, the CI group for the indicated time points had significant muscle damage, manifested by splitting muscle fiber, swelling striated muscle, dissolution, and extensive muscle atrophy (Fig. S1e). The above results indicated that the mice skeletal muscle in the CI group was severely damaged. And the longer the extrusion time, the more serious the damage. Therefore, we successfully constructed the mouse CI model.

**Fig. 1:**
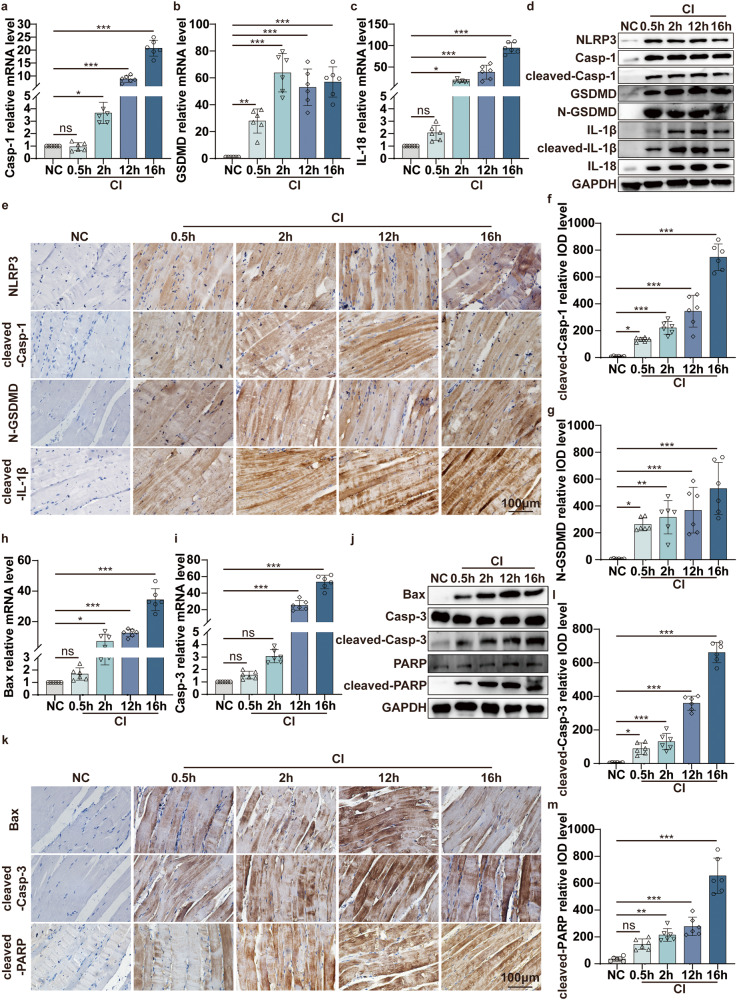
Pyroptosis and apoptosis both occur in the skeletal muscle of the mouse CI model. **a**–**c** qPCR analyses the mRNA level of pyroptosis-associated molecular Casp-1, GSDMD, IL-18 in the skeletal muscle under 1.5 kg pressure for the different indicated crush times (0.5, 2, 12, and 16 h). **d** WB analyses NLRP3, Casp-1, cleaved-Casp-1, GSDMD, N-GSDMD, IL-1β, cleaved- IL-1β, and IL-18 protein expression in the skeletal muscle under different crush times. **e** IHC staining analyses NLRP3, cleaved-Casp-1, N-GSDMD and cleaved-IL-1β protein expression in the skeletal muscle (original magnification: 400×; scale bar: 100 μm). **f**, **g** Quantification of the integrated optical density (IOD) of the image in (**e**). **h**, **i** qPCR analyses the mRNA level of apoptosis-associated molecular Bax and Casp-3 in the skeletal muscle. **j** WB analyses Bax, Casp-3, cleaved-Casp-3, PARP, and cleaved-PARP protein expression in the skeletal muscle. **k** IHC staining analyses Bax, cleaved-Casp-3, cleaved-PARP protein expression in the skeletal muscle (original magnification: 400×; scale bar: 100 μm). **l**, **m** Quantification of the integrated optical density (IOD) of the image in (**k**). One-way ANOVA was used in (**a**–**c**, **f**–**i**, **l**, **m**) (*n* = 6).

Next, we further investigated the underlying molecular mechanisms that lead to the aggravation of skeletal muscle damage in CI. We detected the molecular marker expression of pyroptosis and apoptosis in the indicated crush time points. Compared with the NC group, pyroptosis pathway molecules’ mRNA expression increased with the continuous extension of the crush time in the CI group, including Casp-1, GSDMD, and IL-18 (Fig. 1a–c). WB results showed that the changes in protein levels were consistent with the qPCR results (Fig. 1d). The pyroptosis-associated functional molecules expression was also detected by immunohistochemical staining. The results showed that the effector levels of NLRP3, cleaved-Casp-1, N-GSDMD, and cleaved-IL-1β were obviously increased in the CI group compared with the NC group (Fig. 1e–g). On the other hand, qPCR results showed that compared to the NC group, the apoptosis-associated molecules Bax and Casp-3 expression was increased in the CI group for the indicated 12 h and 16 h (Fig. 1h-i). At the same time, WB results showed that the changes of Bax, cleaved-Casp-3, and cleaved-PARP in protein levels were significantly upregulated from 0.5 h to 16 h in the injured skeletal muscle of CI group (Fig. 1j). Meanwhile, immunohistochemical staining showed the same trend (Fig. 1k–m). Overall, the mRNA of pyroptotic molecules began to have statistical significance around 2 h, while apoptotic molecules are about 12 h. The activated form of pyroptotic effector declined after 16 h, but apoptotic molecules were still at a high level. Whether these results prompted that pyroptosis was initiated slightly before apoptosis in skeletal muscle damage induced by CI remains to be further explored. Taken together, these data supported our hypothesis that both NLRP3/Casp-1/GSDMD-mediated pyroptosis and Casp-3/PARP-mediated apoptosis occur in the skeletal muscle of CI.

### Cell pyroptosis and apoptosis both occur in C2C12 cells of glucose deprivation in vitro ischemic model

**Fig. 2:**
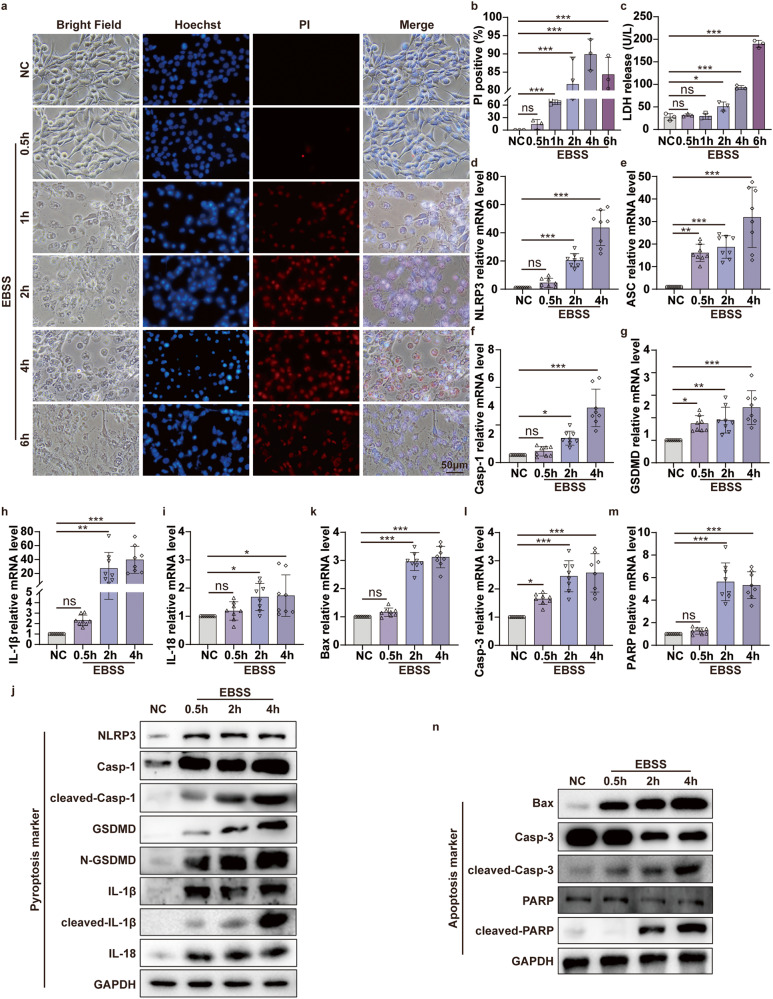
Pyroptosis and apoptosis both occur in C2C12 cells of glucose deprivation in vitro ischemic model. **a** Hoechst 33342/PI double staining to detect pyroptotic C2C12 cells after EBSS treatment for indicated time (0.5, 1, 2, 4, and 6 h). The nuclei are stained blue by Hoechst 33342, and the pyroptotic cells are stained red by PI. Bright-field microscopic images reveal the pyroptotic morphological manifestations of bubble-like formations (original magnification: 400×; scale bar: 50 μm). **b** PI-positive cell proportions. **c** Concentration of LDH in the supernatant. **d**, **i** qPCR analyses the mRNA level of pyroptosis-associated molecular NLRP3, ASC, Casp-1, GSDMD, IL-1β, IL-18 in the C2C12 cells after EBSS treatment for the indicated times (0.5, 2, and 4 h). **j** WB analyses NLRP3, Casp-1, cleaved-Casp-1, GSDMD, N-GSDMD, IL-1β and cleaved-IL-1β, IL-18 protein expression in the C2C12 cells after EBSS treatment. **k**–**m** qPCR analyses the mRNA level of apoptosis-associated molecular Bax, Casp-3, and PARP in the C2C12 cells after EBSS treatment. **n** WB analyses the expression of Bax, Casp3, cleaved-Casp-3, PARP, and cleaved-PARP protein in C2C12 cells after EBSS treatment for indicated times. One-way ANOVA was used in (**b**–**i**, **k**–**m**) (*n* = 8).

To explore the muscle cell death mechanisms in CI, we used an in vitro model of oxygen–glucose deprivation (OGD) to mimic the ischemia and hypoxia situation. We found an interesting phenomenon during the experiment. When C2C12 cells were incubated with EBSS to mimic the glucose deprivation ischemic model, we observed apparent pyroptotic bubbles under phase contrast microscopy (Fig. 2a). With the extension of EBSS incubation time, numbers of propidium iodide (PI)-positive cell and LDH release in the cell supernatant were increased (Fig. 2a–c). In other words, the longer the EBSS incubation time, the more cell damage. Almost all C2C12 cells died when treated with EBSS for 6 h (Fig. 2a). We further examined the expression of pyroptotic molecules. Upon EBSS treatment, the levels of NLRP3, ASC, Casp-1, GSDMD, IL-1β, and IL-18 mRNA were significantly increased in a time-dependent manner in C2C12 cells (Fig. 2d–i). The WB results were consistent with the qPCR results (Fig. 2j). In other words, the activation of pyroptotic effectors (cleaved-Casp-1, N-GSDMD, and cleaved-IL-1β) was increased. Meanwhile, we also detected mainly apoptosis-associated molecules expression. qPCR results showed that Bax, Casp-3, and PARP mRNA was significantly increased in C2C12 cells after EBSS treatment in a time-dependent manner (Fig. 2k–m). The WB results were consistent with the qPCR (Fig. 2n), especially the cleavage of apoptotic effectors Casp-3 and PARP. From the WB results, we also noticed that most of the pyroptotic molecules were activated after EBSS treatment for 0.5 h, but the key apoptotic molecule, cleaved-PARP, was activated after 2 h. This is consistent with previous in vivo results that pyroptosis starts slightly earlier than apoptosis when C2C12 cells are in the ischemia condition. Collectively, these data indicated that the glucose deprivation ischemic model of C2C12 cells coexists with pyroptosis and apoptosis. In addition, from the overall observation results of EBSS-treated C2C12 cells for 0-6 h, the changes in pyroptosis and apoptosis molecules were most significant at 2 h and 4 h. Therefore, we chose 2 h and 4 h incubation times of EBSS for the subsequent glucose deprivation ischemic cell experiments.

### OGD ischemia and hypoxia model significantly alleviate the ischemic-induced cell pyroptosis via NLRP3/Casp-1/GSDMD pathway in vitro

**Fig. 3:**
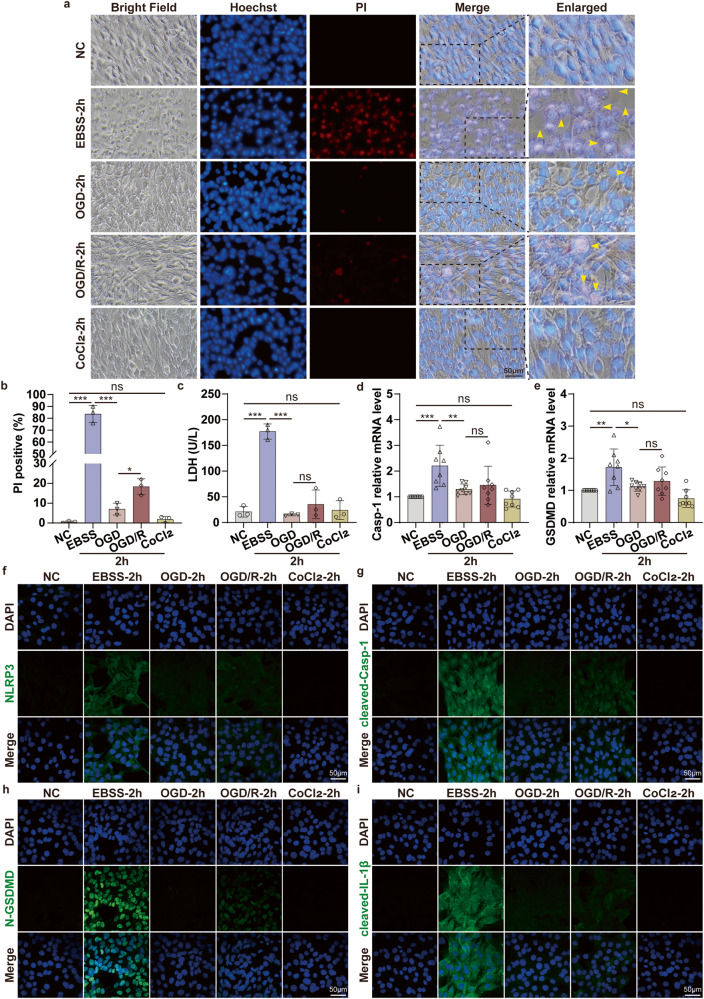
OGD ischemia and hypoxia model significantly alleviate the ischemic-induced cell pyroptosis via NLRP3/Casp-1/GSDMD pathway in vitro. **a** Hoechst 33342/PI double staining to detect pyroptotic C2C12 cells after EBSS (glucose deprivation ischemic model), OGD (oxygen-glucose deprivation ischemia and hypoxia model), OGD/R (after OGD treatment, cells were incubated in a normal condition for 2 h to achieve reperfusion), CoCl_2_ (oxygen deprivation model) treatment for 2 h. Bright-field microscopic images reveal the pyroptotic morphological manifestations of bubble-like formations, indicated by the yellow arrows (original magnification: 400×; scale bar: 50 μm). **b** PI-positive cell proportions. **c** Concentration of LDH in the supernatant. **d**, **e** qPCR analyses the mRNA level of pyroptosis-associated molecular Casp-1, GSDMD in C2C12 cells after EBSS, OGD, OGD/R, and CoCl_2_ treatment. **f**–**i** Representative confocal microscopy images of C2C12 cells in EBSS, OGD, OGD/R, and CoCl_2_ group. The nuclei are stained to blue, and the pyroptosis-associated molecular NLRP3, cleaved-Casp-1, N-GSDMD, and cleaved-IL-1β are stained to green (original magnification: 600×; scale bar: 50 μm). One-way ANOVA was used in (**b**–**e**) (*n* = 8).

Since pyroptosis and apoptosis both occur in the glucose deprivation ischemic C2C12 cells model, what is the situation in the OGD ischemia and hypoxia model? To explore this question, we introduced hypoxia (CoCl_2_) to the in vitro ischemia model to further investigate the changes in pyroptosis and apoptosis-associated molecules. Interestingly, we observed significantly lower PI-positive cells in the OGD group compared to the EBSS group (Fig. 3a, S2a). In other words, the cell survival state was greatly improved, and the pyroptotic bubbles were obviously reduced in the OGD group. Compared to the OGD group, pyroptosis was slightly aggravated after reperfusion in standard medium and normoxic conditions (OGD/R) for 2 h or 4 h (Fig. 3a, S2a). Afterward, we counted the proportion of PI-positive cells in each group (Fig. 3b, S2b) and detected the release of LDH in the cell supernatant (Fig. 3c, S2c). The results showed that the damage degree of C2C12 significantly declined after OGD and OGD/R treatment compared to the EBSS group. Next, we further verified the mechanism of ischemia and hypoxia (OGD group) in decreasing the number of cell death compared with ischemia (EBSS group) treatment in C2C12 cells. qPCR results showed that Casp-1, GSDMD, NLRP3, ASC, IL-1β, and IL-18 mRNA were significantly down-regulated in OGD and OGD/R group compared to the EBSS group (Fig. 3d, e, S2d–m). Congruent with these observations, immunofluorescence showed that pyroptosis key functional molecules expression was significantly downregulated in the OGD group compared to the EBSS group, especially cleaved-Casp-1, N-GSDMD, and cleaved-IL-1β (Fig. 3f–i). Taken together, we found that under the premise of ischemia, oxygen deprivation simultaneously could significantly alleviate the C2C12 cell’s pyroptosis via the NLRP3/Casp-1/GSDMD signaling pathway.

### OGD ischemia and hypoxia model do not alter the ischemic-induced cell apoptosis via the Casp-3/PARP pathway in vitro

**Fig. 4:**
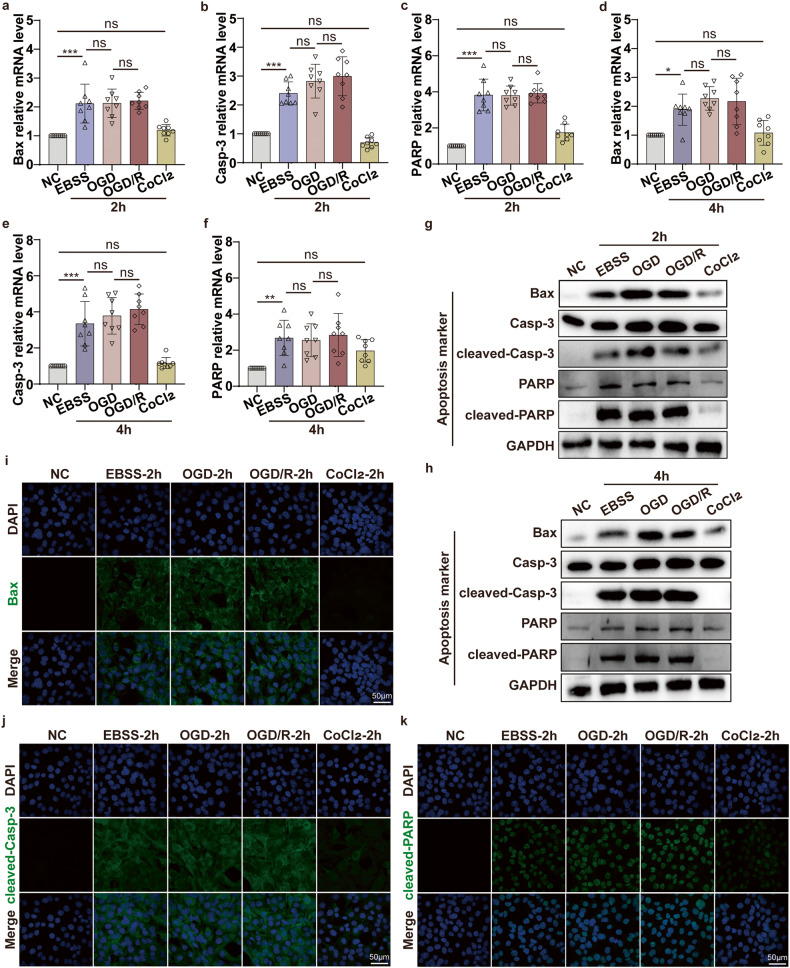
OGD ischemia and hypoxia model does not alter the ischemic-induced cell apoptosis via the Casp-3/PARP pathway in vitro. **a**–**f** qPCR analyses the mRNA level of apoptosis-associated molecular Bax, Casp-3, PARP in the C2C12 cells after EBSS, OGD, OGD/R, CoCl_2_ treatment for 2 h and 4 h. **g**, **h** WB analyses the protein expression level of Bax, Casp-3, cleaved-Casp-3, PARP, and cleaved-PARP in the C2C12 cells after EBSS, OGD, OGD/R, CoCl_2_ treatment for 2 h and 4 h. **i**–**k** Representative confocal microscopy images of C2C12 cells after EBSS, OGD, OGD/R, CoCl2 treatment for 2 h, the nuclei are stained to blue, and the apoptosis-associated molecular Bax, cleaved-Casp-3, cleaved-PARP are stained to green (original magnification: 600×; scale bar: 50 μm). One-way ANOVA was used in (**a**–**f**) (*n* = 8).

Next, we further explored the changes of apoptosis-associated molecules of C2C12 cells in EBSS (ischemia), OGD (ischemia and hypoxia), and OGD/R (ischemia and hypoxia/ reperfusion) group for the indicated time points (2 h or 4 h). qPCR results showed that critical apoptotic molecules Bax, Casp-3, and PARP mRNA in OGD and OGD/R group had no significant changes compared to the EBSS group (Fig. 4a–f). Moreover, WB and IF results showed that the apoptotic effectors Bax, cleaved-casp-3, and cleaved-PARP expression were not altered in the OGD group compared to the EBSS group (Fig. 4g–k). Therefore, the above results indicated that under the premise of ischemia, oxygen deprivation simultaneously did not alter the ischemic-induced cell apoptosis via the Casp-3/PARP pathway in vitro.

Interestingly, OGD ischemia and hypoxia cell model significantly alleviate the ischemic-induced C2C12 cell pyroptosis via NLRP3/Casp-1/GSDMD pathway but do not alter the ischemic-induced cell apoptosis via Casp-3/PARP pathway in vitro. Overall, C2C12 cell damage and cell death were reduced.

### Delayed step-by-step decompression significantly alleviates cell pyroptosis in skeletal muscle of CI mediated by NLRP3/Casp-1/GSDMD pathway

**Fig. 5:**
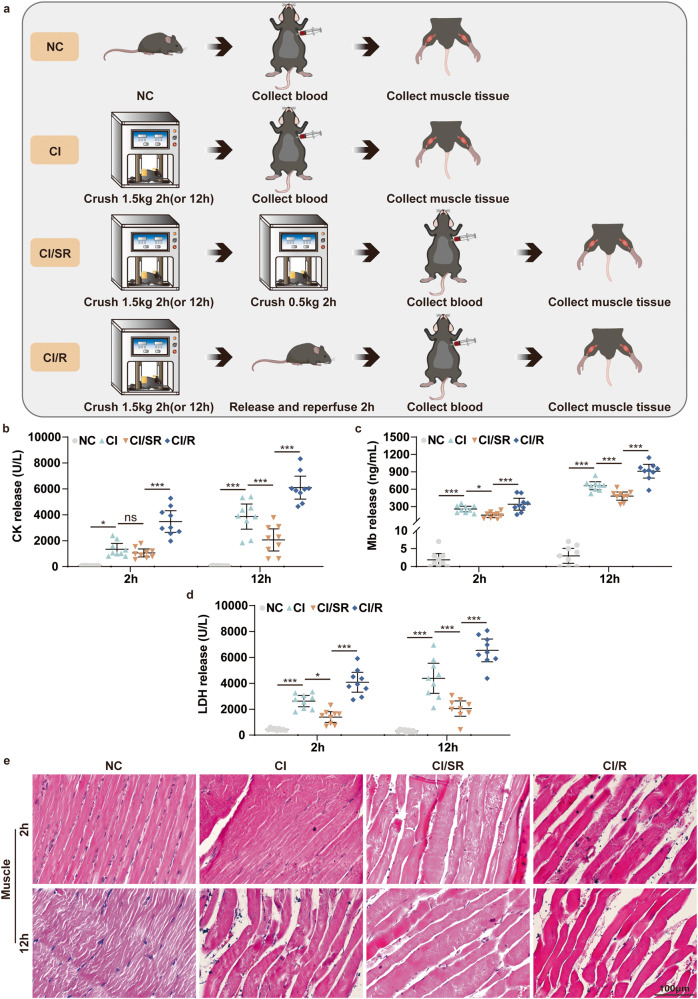
Delayed step-by-step rather than immediate decompression significantly reduces the severity of skeletal muscle in CI. **a** Schematic drawing of the experimental design of delayed step-by-step decompression treatment for CI mice. CI represents that the mouse was crushed under 1.5 kg for 2 h or 12 h. CI/SR represents that the mouse was crushed under 1.5 kg for 2 h or 12 h, then maintained squeezed under 0.5 kg pressure for an additional 2 h. CI/R represents that the mouse was crushed under 1.5 kg for 2 h or 12 h, then directly decompression and reperfusion for 2 h. **b**–**d** CK, Mb, and LDH concentration in serum. **e** HE staining analyses skeletal muscle pathological changes under different crush conditions for 2 h and 12 h (NC, CI, CI/SR, CI/R) (original magnification: 400×; scale bar: 100 μm). Two-way ANOVA was used in (**b**–**d**) (*n* = 9).

Through the previous in vitro experiments, we found that under the premise of muscle cell ischemia, reducing the oxygen concentration could alleviate muscle cell damage. So, we wondered, could delayed step-by-step decompression of CI patients that slow down the reperfusion process minimize muscle injury and cell death? To test our hypothesis, we set up four experimental groups, namely NC, CI, CI/SR (slow decompression and reperfusion), CI/R (direct decompression and reperfusion), and selected two crush times of 2 h and 12 h according to the previous study (Fig. 5a). The results of serum biochemistry analysis showed that compared with the NC group, the CK (Fig. 5b), Mb (Fig. 5c), and LDH (Fig. 5d) in the CI group were significantly increased. More importantly, compared to the CI group, the concentration of serum CK, Mb, and LDH significantly decreased in CI/SR group (Fig. 5b–d). Meanwhile, direct reperfusion increased the expression of the above molecules in the CI/R group. HE staining showed that compared to the NC group, the muscle tissue of the CI group and the CI/R group at 12 h was severely damaged, with swelling muscle, ruptured muscle fiber, and infiltration of inflammatory cells (Fig. 5e). In contrast, the muscle tissue damage in the CI/SR group was significantly lighter, and the muscle fibers were still dense and ordered. These results suggested that delayed step-by-step decompression rather than the immediate release of all pressure can dramatically reduce the severity of skeletal muscle damage in CI.

**Fig. 6:**
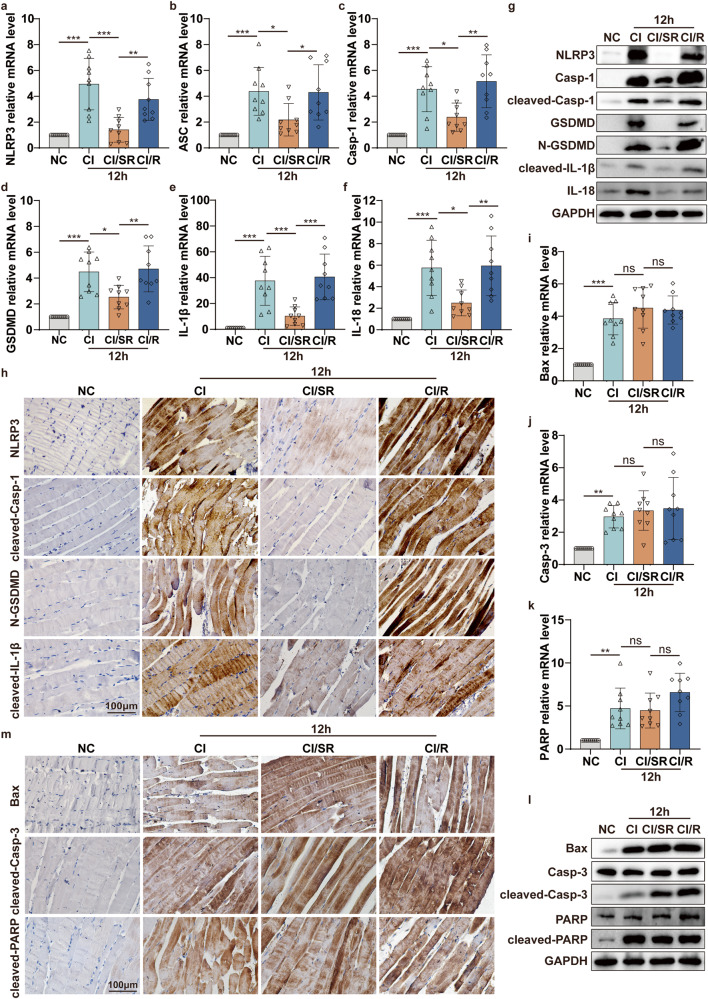
Delayed step-by-step decompression significantly alleviates cell pyroptosis in skeletal muscle of CI mediated by NLRP3/Casp-1/GSDMD pathway. **a**–**f** qPCR analyses the mRNA level of pyroptosis-associated molecular NLRP3, ASC, Casp-1, GSDMD, IL-1β, IL-18 in the skeletal muscle of mice crushed for 12 h followed by different decompression methods (NC, CI, CI/SR, CI/R). **g** WB analyses the protein expression level of NLRP3, Casp-1, cleaved-Casp-1, GSDMD, N-GSDMD, cleaved-IL-1β and IL-18 in the skeletal muscle tissues of different groups. **h** IHC staining analyses the representative pyroptotic molecules NLRP3, cleaved-Casp-1, N-GSDMD, and cleaved-IL-1β protein expression in skeletal muscle tissues of different groups (original magnification: 400×; scale bar: 100 μm). **i**–**k** qPCR analyses the mRNA level of apoptosis-associated molecular Bax, Casp-3, and PARP in the skeletal muscle of mice crushed for 12 h followed by different decompression methods (NC, CI, CI/SR, CI/R). **l** WB analyses Bax, Casp-3, cleaved-Casp-3, PARP, and cleaved-PARP protein expression in the skeletal muscle tissues of different groups. **m** IHC staining analyses the representative apoptotic molecules Bax, cleaved-Casp-3, and cleaved-PARP protein expression in skeletal muscle tissues of different groups (original magnification: 400×; scale bar: 100 μm). One-way ANOVA was used in (**a**–**f**, **i**–**k**) (*n* = 9).

Inspired by this exciting discovery, then we detected pyroptosis-associated molecules expression. qPCR results indicated that the mRNA levels of pyroptosis-associated molecules in the CI/SR group were significantly lower than those in the CI/R group, even lower than the CI group (Fig. 6a–f, S3a–f). Consistent with the qPCR analysis, the protein expression levels of NLRP3, Casp-1, GSDMD, and IL-18, especially pyroptotic effectors cleaved-casp-1, N-GSDMD, cleaved-IL-β were significantly decreased in CI/SR group compared to CI or CI/R group (Fig. 6g, h, S3g, h). Taken together, the above results indicated that during the decompression process of CI mice, the delayed step-by-step decompression could significantly inhibit skeletal muscle pyroptosis via NLRP3/Casp-1/GSDMD signaling pathway.

### Delayed step-by-step decompression does not alter cell apoptosis in the skeletal muscle of CI mediated by the Casp-3/PARP pathway

Moreover, we also detected the changes in apoptosis-related molecules in the skeletal muscle of each group. qPCR results showed that under the condition of 1.5 kg extrusion for 12 h, the mRNA levels of apoptotic molecules Bax, Casp-3, and PARP were no changes among CI, CI/SR, and CI/R groups (Fig. 6i–k). Meanwhile, the apoptotic effectors Bax, cleaved-Casp-3, and cleaved-PARP expression were not altered in CI/SR group compared to the CI group but showed a slight decrease compared to the CI/R group (Fig. 6l, m). Meanwhile, we observed the same results in the short crush time (2 h) of the mice CI model (Fig. S4a–e). Therefore, during the decompression process of CI mice, the delayed step-by-step release pressure does not alter skeletal muscle apoptosis via the Casp-3/PARP pathway.

Overall, we found that delayed step-by-step decompression of CI mice could alleviate skeletal muscle injury by inhibiting cell pyroptosis via NLRP3/Casp-1/GSDMD pathway but not cell apoptosis via the Casp-3/PARP pathway.

### Delayed step-by-step decompression and/or administration of the pyroptotic inhibitor DSF alleviates skeletal muscle and kidney injury of CS-AKI mice induced by CI

**Fig. 7:**
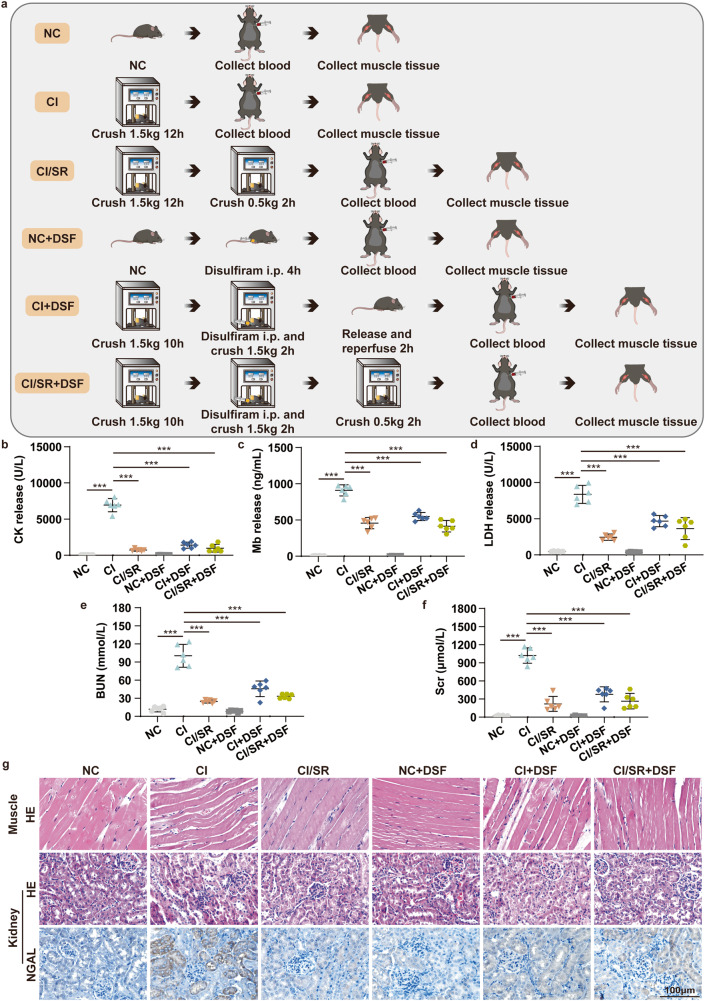
Delayed step-by-step decompression and/or pyroptotic inhibitor therapy alleviates skeletal muscle and kidney injury of CS-AKI mice induced by CI. **a** Schematic drawing of the experimental design of the treatment of CI mice. CI represents that the mouse was crushed under 1.5 kg for 12 h. CI/SR represents that the mouse was crushed under 1.5 kg for 12 h, then maintained squeezed under 0.5 kg pressure for an additional 2 h. NC + DSF represents that mouse was intraperitoneal injection DSF for 4 h. CI + DSF represents that mouse was crushed under 1.5 kg for 10 h, administration of DSF, and maintained at 1.5 kg for an additional 2 h before direct decompression and reperfusion for 2 h. CI/SR + DSF represents that mouse was crushed under 1.5 kg for 10 h, administration of DSF, and maintained at 1.5 kg for an additional 2 h before maintained squeezed under 0.5 kg pressure for an additional 2 h. **b**–**f** CK, Mb, LDH, BUN, and Scr concentration in serum. **g** HE staining analyses skeletal muscle and kidney pathological changes under different crush conditions for 12 h (NC, CI, CI/SR, NC + DSF, CI + DSF, CI/SR + DSF). IHC detected NGAL expression in the kidney of different groups (NC, CI, CI/SR, NC + DSF, CI + DSF, CI/SR + DSF) (original magnification: 400×; scale bar: 100 μm). One-way ANOVA was used in (**b**–**f**) (*n* = 6).

Considering that delayed step-by-step decompression of CI mice could alleviate the skeletal muscle injury by inhibiting cell pyroptosis via NLRP3/Casp-1/GSDMD, so we wonder if the treatment with pyroptotic inhibitors during the rescue process can achieve a similar therapeutic effect. To answer this conjecture, mice are divided into 6 groups: NC, CI, CI/SR, NC + DSF (administration of DSF) group, CI + DSF (administration of DSF then directly decompression and reperfusion), CI/SR + DSF (administration of DSF then slowly decompression) (Fig. 7a).

Compared with the NC group, the blood biochemical indicators CK, Mb, BUN, and Scr of the CI group, as well as the results of HE staining and NGAL molecular immunohistochemistry staining of kidney tissue, showed that we also successfully constructed a mouse model of CS-AKI induced by CI (Fig. 7b, c, e–g). Compared with the CI group, the contents of CK, Mb, LDH, BUN, and Scr in the serum of the CI + DSF group were significantly decreased (Fig. 7b–f), and muscle and kidney damage was also considerably reduced (Fig. 7g). In other words, administering pyroptotic inhibitors before direct decompression can reduce muscle and kidney damage in CS-AKI mice caused by CI. Meanwhile, we found that the therapeutic effect of DSF was similar to delayed step-by-step decompression (Fig. 7b–g). Overall, pyroptotic inhibitor therapy alone, delayed step-by-step decompression, or combined with delayed step-by-step decompression and pyroptotic inhibitor therapy significantly alleviated muscle and kidney damage in CS-AKI mice caused by CI.

**Fig. 8:**
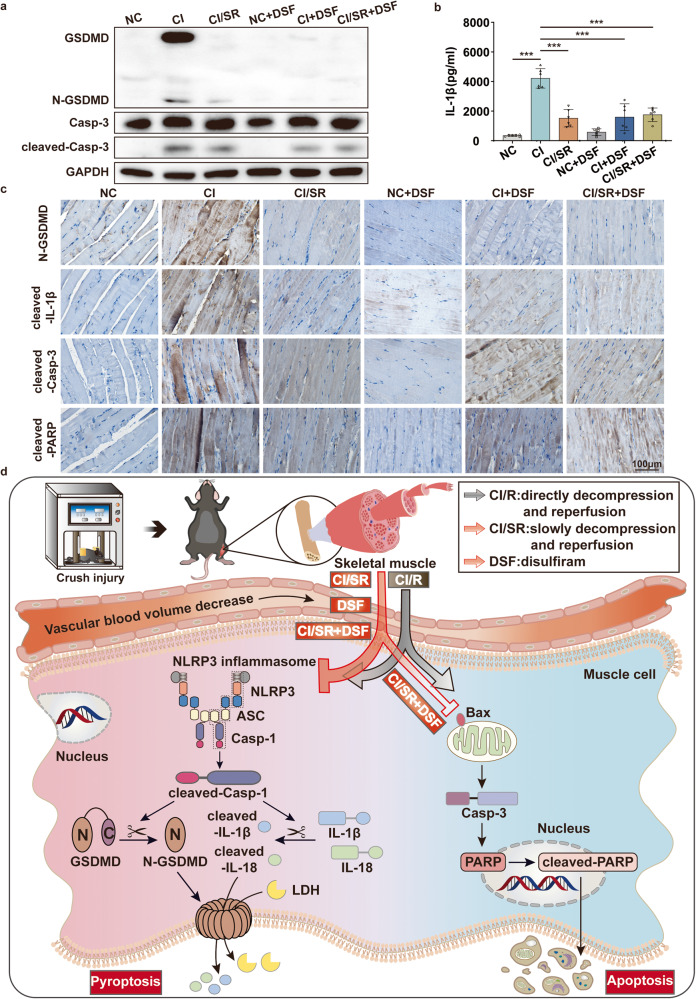
The synergism of delayed step-by-step decompression and pyroptotic inhibitor DSF therapy reduce pyroptosis and apoptosis-associated molecules expression of skeletal muscle in CI. **a** WB analyses GSDMD, N-GSDMD, Casp-3, cleaved-Casp-3 protein expression in the skeletal muscle under different groups. **b** ELISA analyses IL-1β expression in serum. **c** IHC staining analyses the representative effectors of pyroptotic (N-GSDMD, cleaved-IL-1β) and apoptotic (cleaved-Casp-3, cleaved-PARP) in skeletal muscle tissues of different groups (original magnification: 400×; scale bar: 100 μm). One-way ANOVA was used in (b) (n = 6). **d** Schematic diagram of cell pyroptosis and apoptosis mechanisms during injured skeletal muscle decompression in CI. In the skeletal muscle of CI, I/R injury caused by direct decompression (CI/R) will seriously damage skeletal muscle through NLRP3/Casp-1/GSDMD pyroptosis signaling pathway and the Casp-3/PARP apoptosis signaling pathway. However, delayed step-by-step decompression or pyroptotic inhibitor DSF significantly alleviates skeletal muscle damage by inhibiting the NLRP3/Casp-1/GSDMD pyroptosis pathway but not altering the Casp-3/PARP apoptosis pathway. Moreover, the synergism of delayed step-by-step decompression and DSF therapy reduce the activation of pyroptosis and apoptosis pathways in the skeletal muscle of CI.

What is the mechanism of muscle damage mitigation? WB, immunohistochemistry, and enzyme-linked immunosorbent assay (ELISA) results showed that compared with the CI group, the expression of pyroptotic executioner N-GSDMD and the pro-inflammatory cytokine cleaved-IL-1β was significantly reduced in the CI/SR, CI + DSF, CI/SR + DSF group (Fig. 8a–c). Meanwhile, we found that the apoptotic effector cleaved-Casp-3 and cleaved-PARP were also slightly reduced in the CI/SR + DSF group compared to CI/SR or CI group (Fig. 8a, c). Therefore, the combination of delayed step-by-step decompression and pyroptotic inhibitor DSF therapy may become a novel and effective treatment for patients with CI, even CS-AKI.

## Discussion

Previous research indicated that autophagy and necrosis represented extremes of the muscle response to injury [26]. Meanwhile, inflammation plays a vital role in the pathophysiology of skeletal muscle injury [27, 28]. However, whether the process of CI is accompanied by pyroptosis (pro-inflammation) and apoptosis of skeletal muscle cells and the underlying mechanism remain unclear, and need to be further explored.

In this study, we confirmed that pyroptosis and apoptosis both occur in the skeletal muscle of mice CI. Maybe pyroptosis occurred slightly earlier than apoptosis. Interestingly, we found that glucose deprivation ischemic C2C12 cells model could occur pyroptosis. However, under the premise of ischemia, if the oxygen was further deprived (oxygen–glucose deprivation, OGD), the damage degree of C2C12 cells was reduced. This is consistent with the idea that hypoxia considers being the fundamental pathology for I/R diseases [29]. Meanwhile, Ryan et al. indicated that ischemic post-conditioning is most effective for the salvage of skeletal muscle I/R injury between 4 h and 6 h [30]. Therefore, we speculate that in the process of skeletal muscle decompression of CI, if the blood oxygen content around the ischemic tissue is controlled, is it possible to alleviate muscle damage by affecting the pyroptosis and apoptosis of muscle cells? To answer this question, we further confirmed that delayed step-by-step decompression of CI mice could significantly reduce skeletal muscle injury by inhibiting the NLRP3/Casp-1/GSDMD pyroptosis pathway but not altering the Casp-3/PARP apoptosis pathway. Moreover, pyroptotic inhibitor therapy has a similar effect. And the combination of delayed step-by-step decompression and pyroptotic inhibitor therapy not only significantly decreases pyroptosis-related molecules expression but also slightly reduces apoptosis-related molecules expression. Overall, compared with CI or CI/R group, the effect of delayed step-by-step decompression of CI mice (CI/SR group), or CI + DSF group or CI/SR + DSF group obviously reduced the skeletal muscle and kidney injury. Our research provides a new way of thinking about the plight of CI or CS-AKI casualties.

PCD is a fundamental physiological process in all living organisms [31]. The most studied forms of cell death at this stage include apoptosis, necroptosis, pyroptosis, autophagy, ferroptosis, and copper death [32, 33]. Pyroptosis is caused by the activation of so-called inflammasome sensors, ultimately resulting in the loss of plasma membrane integrity [34]. Morphological features of pyroptosis include bubble-like protrusions emersion, plasma membrane pores formation, cell swelling, membrane rupture, and inflammatory mediators release such as IL-1β and IL-18 [35]. Unlike pro-inflammatory pyroptosis, apoptosis is characterized by activated initiation caspase-8, -9, and -10, and effector caspase-3, -6, and -7 [32]. Apoptosis ultimately leads to disruption of the nuclear envelope, cleavage of many intranuclear proteins such as PARP and lamin, and formation of apoptotic bodies [36].

The skeletal muscle is the largest cellular compartment of the body and acts as an immunologically unique tissue. Recently research indicated that recognition and clearance of dying cells in the skeletal muscle are vital for muscle homeostasis and tolerance maintenance [26]. In addition to PCD under physiological conditions, skeletal muscle cell death is a sign of some muscle diseases. Nazir et al. indicated that compared to apoptosis, pyroptosis is more essential and needs early intervention in the myocardial ischemic injury of mice. Because pyroptosis can occur within 1 h, but apoptosis often occurs after 12 h [37]. Similar to myocardial ischemic injury, our results confirm that apoptosis and pyroptosis both occur in the injured skeletal muscle of mice CI. And the occurrence of pyroptosis and apoptosis in the injured skeletal muscle induced by CI may be sequential, and pyroptosis seems slightly earlier than apoptosis.

The NLRP3 inflammasome assembles upon sensing stimuli and activates Casp-1 leading to cleavage of GSDMD, which triggers pyroptosis by forming membrane pores and up-regulating pro-inflammatory mediator secretion [38]. Recent research has shown that NLRP3 inflammasome is activated in multiple animal models of skeletal muscle injury [39, 40]. Consistent with our findings, skeletal muscle damage of CI induced by heavy object crushing of both hindlimbs in mice promotes muscle cell pyroptosis by activating NLRP3 and cleaved-Casp-1, cleaving GSDMD, and releasing IL-18 and IL-1β. The massively released IL-1β and IL-18 further amplify the inflammatory cascade by promoting additional chemokines, cytokines, and adhesion molecules. Therefore, we speculate that this inflammatory cascade triggered by muscle pyroptosis at the crush site is a vital cause of systemic inflammatory response and multiple organ damage. Overall, this study verified that using pyroptotic inhibitor DSF suppressed pyroptosis of skeletal muscle and the skeletal muscle and kidney injury was also alleviated.

The muscle tissue is squeezed for a long time by heavy objects, and the muscle cells die due to the ischemic and hypoxic conditions. Large amounts of Mb and potassium ions in rupture muscle cells are confined locally. But when the buried person is rescued, the pressure on the muscles is suddenly lifted, blood circulation is restored, and a large influx of dangerous molecules into the bloodstream causes reperfusion injury. So, when the wounded are lifted out of the rubble, it doesn’t mean the end of the rescue effort or even the danger has just begun. I/R injury has been identified as the primary pathogenic mechanism of muscle injury, especially myocardial I/R injury [41, 42]. However, the injury mechanism and process of skeletal muscle in CI are complex and unclear. Our investigation found an interesting phenomenon. In the ischemic condition, in other words, glucose deprivation ischemic C2C12 cells could occur pyroptosis. However, if the oxygen was further deprived (OGD), the pyroptosis signalling pathway was significantly reduced, and the apoptosis pathway was not altered. Overall, PI-positive cell numbers decreased. This is consistent with Zhao’s research that ischemic post-conditioning during reperfusion inhibits myocardial injury [43]. Ischemic post-conditioning may delay the washout of endogenous DAMPs. The above researches support the idea that hypoxia considers being the fundamental pathology for I/R diseases [29].

Inspired by this phenomenon, we speculated that delayed step-by-step decompression of CI, in other words, slowly increasing blood flow and oxygen levels of the ischemia skeletal muscle tissue, will be better for the patients during the rescue. Results supported our conjecture that delayed step-by-step decompression could significantly alleviate skeletal muscle injury of mice CI by inhibiting the NLRP3/Casp-1/GSDMD pyroptosis pathway but not altering the Casp-3/PARP apoptosis pathway. In addition, we found that pyroptotic inhibitor DSF has similar therapeutic effects. Meanwhile, the combination of delayed step-by-step decompression and pyroptotic inhibitor therapy has a better effect. Because it not only significantly suppresses the activation of pyroptotic effectors but also slightly decreases the activation of apoptotic effectors. At the same time, Fu et al. indicated that exogenous carbon monoxide (CORM-3) protects against mitochondrial DNA‑induced hippocampal pyroptosis in rat models of hemorrhagic shock and resuscitation [44]. Zhang et al. found that CORM-3 alleviated neuronal pyroptosis and improved neurological recovery in hemorrhagic shock and resuscitation through mitochondrial regulation mediated by the sGC-cGMP pathway [45]. The above results supported our findings from the side. It is necessary to slowly increase the oxygen concentration and inhibit pyroptosis in the injured skeletal muscle of CI. The new physical stress relief and drug intervention method proposed in this study put forward new ideas and directions for the rescue of patients with CI, even CS-AKI.

Although our current research has preliminarily proved that releasing pressure in steps, slowly restoring blood supply and oxygen, could weaken the reperfusion injury of CI. However, there are still some shortcomings in our research. Firstly, only 0.5 kg intermediated pressure value was used in delayed step-by-step decompression progress, and it is necessary to refine the extrusion pressure further and explore the influence of delayed step-by-step pressure release on skeletal muscle pyroptosis and apoptosis in detail. Secondly, this study only focused on the effects of ischemia, hypoxia, and reperfusion on pyroptosis and apoptosis. But did not do in-depth research on hypoxic conditions alone and the underlying mechanisms among oxidative stress, pyroptosis and apoptosis. Some studies have shown that CoCl_2_ treatment for different times (more than 24 h) can lead to varying degrees of pyroptosis, apoptosis, necrosis, and autophagy of C2C12 cells [46, 47]. But in our study, CoCl_2_ was incubated with C2C12 cells only for a short time (2 h and 4 h). And the comparison of the results between the NC group and the CoCl_2_ group showed that short-term hypoxia alone had a slight tendency to induce apoptosis, suggesting that the toxic effects of CoCl_2_ on C2C12 cells may be strictly limited by the treatment time [48]. DSF acts as a pyroptotic inhibitor. It inhibits the membrane pores formation caused by N-GSDMD but does not break down the pores that have already formed [49]. The upstream molecules of the pyroptosis signalling pathway remain and need to be studied. In the future, CRISPR-Cas9 technology and gene knockout mice should also be used to silence key molecules of pyroptosis and apoptosis so as to conduct more accurate verification and exploration at the gene level.

Taken together, our work suggests a mechanism for alleviating skeletal muscle damage by significantly inhibiting the NLRP3/Casp-1/GSDMD pyroptosis pathway but not altering the Casp-3/PARP apoptosis pathway in delayed step-by-step decompression of CI mice. Meanwhile, pyroptotic inhibitor DSF alone, or the combination of delayed step-by-step decompression and DSF, can both alleviate muscle and kidney injury. In other words, our data identify a novel potential rescue or therapeutic strategy for patients with skeletal muscle injury induced by CI, even CS-AKI. When the heavy objects on the crushed injured body are relieved, a delayed step-by-step relief method is adopted to ease the blood supply of the injured muscle. Pyroptosis-targeted intervention strategies for treating CI-related pathologies can effectively alleviate tissue damage. This may indicate a therapeutic application, which is worth further investigation. Overall, this study points out the direction for the rescue dilemma of CI or CS-AKI and provides a new rescue idea and measures.

## Materials and methods

### Animal models

Adult Male C57BL/6J mice (8–10 weeks old, about 20 g) were housed in a specific pathogen-free husbandry with 12 h light/dark cycles and had free access to food and water. All mice were randomly divided into different groups. First, we allocated a batch of mice into the control group (NC) and CI group. Mice in the NC group did not receive crush treatments. For preparing the mouse CI model, after anesthesia, the digital crush platform [50] was used to continuously crush both hind limbs of mice under a pressure of 1.5 Kg [51] for 0.5, 2, 12, and 16 h, respectively. Mice were sacrificed immediately after decompression, and blood was collected for serum biochemistry. The muscle tissues were quickly removed for western blotting and qPCR or stored at 4% paraformaldehyde for hematoxylin-eosin (HE) and immunohistochemistry staining. For subsequent animal CI rescue experiments, we divided mice into NC group, CI group (1.5 kg squeezed for 2 h or 12 h), CI/SR group (1.5 kg squeezed for 2 h or 12 h, then maintained squeezed under 0.5 kg pressure for additional 2 h), CI/R (1.5 kg squeezed for 2 h or 12 h, directly decompression, and reperfusion for 2 h), NC + DSF (administration of DSF for 4 h), CI + DSF (1.5 kg squeezed for 12 h, administration of DSF for 2 h before directly decompression and reperfusion for 2 h), CI/SR + DSF (1.5 kg squeezed for 12 h, and administration of DSF for the last 2 h, then maintained squeezed under 0.5 kg pressure for additional 2 h), their blood and muscle tissues of each group were collected. Investigators were not blinded to group allocation during the experiment or when analyzing the outcome.

### Cell culture and treatment

Mouse C2C12 cells (ATCC) were cultured in DMEM-high glucose medium containing 10% fetal bovine serum and antibiotics (100 IU/mL penicillin and 1 mg/mL streptomycin) with a humidified atmosphere containing 5% CO_2_ at 37 °C. Authentication and mycoplasma test were confirmed by the supplier, and morphological examination was performed by light microscopy. C2C12 cells incubated with glucose-free Earle’s Balanced Salt Solution (EBSS, 6800 mg/L sodium chloride (NaCl), 2200 mg/L sodium bicarbonate (NaHCO_3_), 122 mg/L sodium phosphate monobasic (NaH_2_PO4), 400 mg/L potassium chloride (KCl)) for 0.5, 1, 2, 4, and 6 h respectively to mimic glucose deprivation ischemic model [52]. For the OGD ischemia and hypoxia model, 200 µM Cobalt chloride (CoCl_2_) was added for oxygen deprivation [53, 54] at the same time as EBSS treatment for the indicated time. For the OGD/R group, the OGD medium was replaced with a standard culture medium after OGD treatment. Then cells were incubated in a normal condition for 2 h to achieve reperfusion [55]. For the CoCl_2_ group, added 200 µM CoCl_2_ to the standard medium for the indicated time.

### Biochemistry analysis

The mouse blood samples were centrifuged for 15 min at 3000 rpm. Serum was collected into 1.5 mL centrifuge tubes (NEST Biotechnology Co. Ltd.). The levels of Mb, CK, and LDH in serum and C2C12 cell culture supernatants were detected by an automatic biochemical analysis instrument (iMagic-V7, Icubio).

### Hematoxylin–eosin (HE)

The isolated muscle tissue samples were fixed in 4% paraformaldehyde. Then the tissue was dehydrated and embedded in paraffin and sectioned into 4 μm thickness. After staining with HE and dehydration, an optical microscope was used to observe and evaluate the pathological changes in skeletal muscle tissue.

### Immunohistochemistry (IHC) and immunofluorescence (IF)

The muscle sections were deparaffinized with xylene, rehydrated with alcohol, incubated with 3% hydrogen peroxide, and heated for antigen repair. Plasma membranes were permeabilized with 0.5% Triton X-100. 5% goat serum (Beijing Solarbio Science & Technology Co., Ltd., #SL038) was used to block non-specific binding sites. Sections were then incubated with primary antibodies against NLRP3 (1:100, Santa Cruz, sc-134306), N-GSDMD (1:200, CST, #36425), cleaved-Casp-1 (1:200, CST, #4199), cleaved-IL-1β (1:300, Affinity, #AF4006), Bax (1:300, CST, #14796), cleaved-Casp-3 (1:200, Affinity, #AF7022), cleaved-PARP (1:200, CST, #5625). Then the sections were incubated with HRP-coupled secondary antibodies at room temperature. After washing, the sections were stained with DAB (Beijing Solarbio Science & Technology Co., Ltd., #DA1015), and the nuclei were re-stained with hematoxylin. After mounting with neutral gum, the muscle tissue sections were observed and photographed. For the immunofluorescence, cell slides were fixed with 4% paraformaldehyde. The plasma membrane was permeated with 0.5% Triton X-100, and non-specific binding sites were blocked with 5% BSA (Beijing Solarbio Science & Technology Co., Ltd., #A8010). After being incubated with primary antibodies against NLRP3, N-GSDMD (1:100), cleaved-Casp-1 (1:100), cleaved-IL-1β (1:100), Bax, cleaved-Casp-3, cleaved-PARP at 4 °C overnight. Then incubated with secondary antibodies coupled to Alexa Fluor 488 (1:200, ZSGB-BIO, #ZF-0511, and #ZF-0512) for 1 h. The cell nuclei were stained by 4’,6-diamidino-2-phenylindole (DAPI) (Beijing Solarbio Science & Technology Co., Ltd., #C0060) in the dark. At last, the slides were observed and photographed by confocal microscopy (Nikon, A1).

### Quantitative real-time PCR (qPCR)

Muscle tissue and C2C12 cells were lysed. And total RNA was extracted by TRIeasy™ LS Total RNA Extraction Reagent (YEASEN, #19201ES60) according to the manufacturer’s instructions. The purity and concentration of the extracted RNA samples were determined using Nanodrop One. Reverse transcription into cDNA was performed using the Hifair® II 1st Strand cDNA Synthesis Kit (YEASEN, #11141ES60). Then Hieff® qPCR SYBR Green Master Mix (No Rox) (YEASEN, #11201ES03) kit was used to perform qPCR with LightCycler® 96 instrument (Roche). The 2^−^^ΔΔCt^ method was used to calculate relative expression levels. The primers are listed in Table S1.

### Western blotting (WB)

Proteins were extracted from muscle tissue and C2C12 cells using ice-cold RIPA lysis buffer containing protease and phosphatase inhibitors. The samples were quantified with the BCA protein quantification kit (YEASEN, #20201ES76). Total protein (30 μg) was separated by SDS-PAGE and subsequently transferred onto PVDF membranes. After blocking with 5% skimmed milk (Shandong Sparkjade Biotechnology Co., Ltd., #ED0019), then the membranes were incubated with primary antibodies against NLRP3 (1:500), Casp-1(1:2000, Affinity, #AF5418), cleaved-Casp-1 (1:2000), GSDMD (1:2000, Affinity, #AF4012), N-GSDMD (1:2000), IL-18 (1:2000, Affinity, #DF6252), IL-1β (1:2000, Affinity, #AF5103), cleaved-IL-1β (1:2000), Bax (1:2000), Casp-3 (1:2000, CST, #9662), cleaved-Casp-3 (1:2000), PARP (1:2000, CST, #9542), and GAPDH (1:5000, Sungene Biotech, #KM9002T; 1:5000, Beijing Solarbio Science & Technology Co., Ltd., #K200103M) overnight at 4 °C on a shaker. Then the membranes were incubated with HRP-coupled secondary antibodies (1:5000, Sungene Biotech, #LK2001, #LK2003) on a shaker. The Enhanced ECL Chemiluminescent Substrate Kit (YEASEN, #36222ES60) was used to visualize protein bands, and Tanon 5200 Multiple Detection System was used for imaging. The intensity value of bands was analyzed using the Tanon Gel-Pro Analyzer system.

### Hoechst 33342/PI dual staining

Hoechst 33342 and PI double staining was used to identify the pyroptotic C2C12 cells as described previously [56, 57]. At the end of all interventions, the cells were fixed with 4% paraformaldehyde and then followed by Hoechst 33342 (blue)/PI (red) (Beijing Solarbio Science & Technology Co., Ltd., # CA1120) staining solution for 30 min at 4 °C in the dark according to the manufacturer’s instruction. The stained images were observed by fluorescence microscope (Nikon, Ts2R).

### ELISA

IL-1β content in mouse serum and cell culture supernatant was determined using Mouse IL-1β ELISA KIT ((Beijing Solarbio Science & Technology Co., Ltd., # SEKM-0002). According to the instructions of the kit, 100 μl standard sample and test sample were put into the reaction hole and incubated at 37 °C for 90 min. Then added, 100 μl biotinylated antibody and enzyme conjugate working solution, respectively, and incubated at 37 °C for 60 min and 30 min. Next, 100 μl chromogenic substrate was added and incubated at 37 °C in the dark for 15 min. Finally, added 50 μl termination solution and immediately measured the OD value with the microplate reader (Hangzhou Allsheng Instruments Co., Ltd., FlexA-200) at 450 nm wavelength.

### Statistical analysis

Each experiment was repeated at least three times, and continuous variables with normal distribution were presented as mean ± standard deviation. For more than two groups, one-way ANOVA or two-way ANOVA followed by Tukey’s multiple comparisons test were used to identify the differences. Underlying assumptions for these tests, including sample independence, variance equality, and normality, were assumed to be met. The sample size conforms to standard protocols in the field. All measurements were taken from different samples. Mice were excluded from experiments if they died. No data were excluded. Three levels of statistical significance (*P < 0.05; **P < 0.01; ***P < 0.001; ns: no significance.) were set. *P* < 0.05 was considered statistically significant. All data statistical analysis and image generation were performed in GraphPad Prism software. Test methods used to assess significance are detailed in each figure caption.

## Supplementary information

Supplementary information

Original full length western blots

## Data Availability

All data that support the findings of this study are available from the corresponding authors upon reasonable request.
