# Supplementary material for: Delayed step-by-step decompression with DSF alleviates skeletal muscle crush injury by inhibiting NLRP3/CASP-1/GSDMD pathway
Source: Cell Death Discov. 2023 Aug 1;9:280. doi: 10.1038/s41420-023-01570-3 (PMC10394048; doi:10.1038/s41420-023-01570-3)
Supplement: Supplementary file 1 — Supplementary information [file 41420_2023_1570_MOESM1_ESM.docx]

**Supplementary Information for**

**Delayed step-by-step decompression with DSF alleviate skeletal muscle crush injury by inhibiting NLRP3/CASP-1/GSDMD pathway**

**Supplementary Figures**


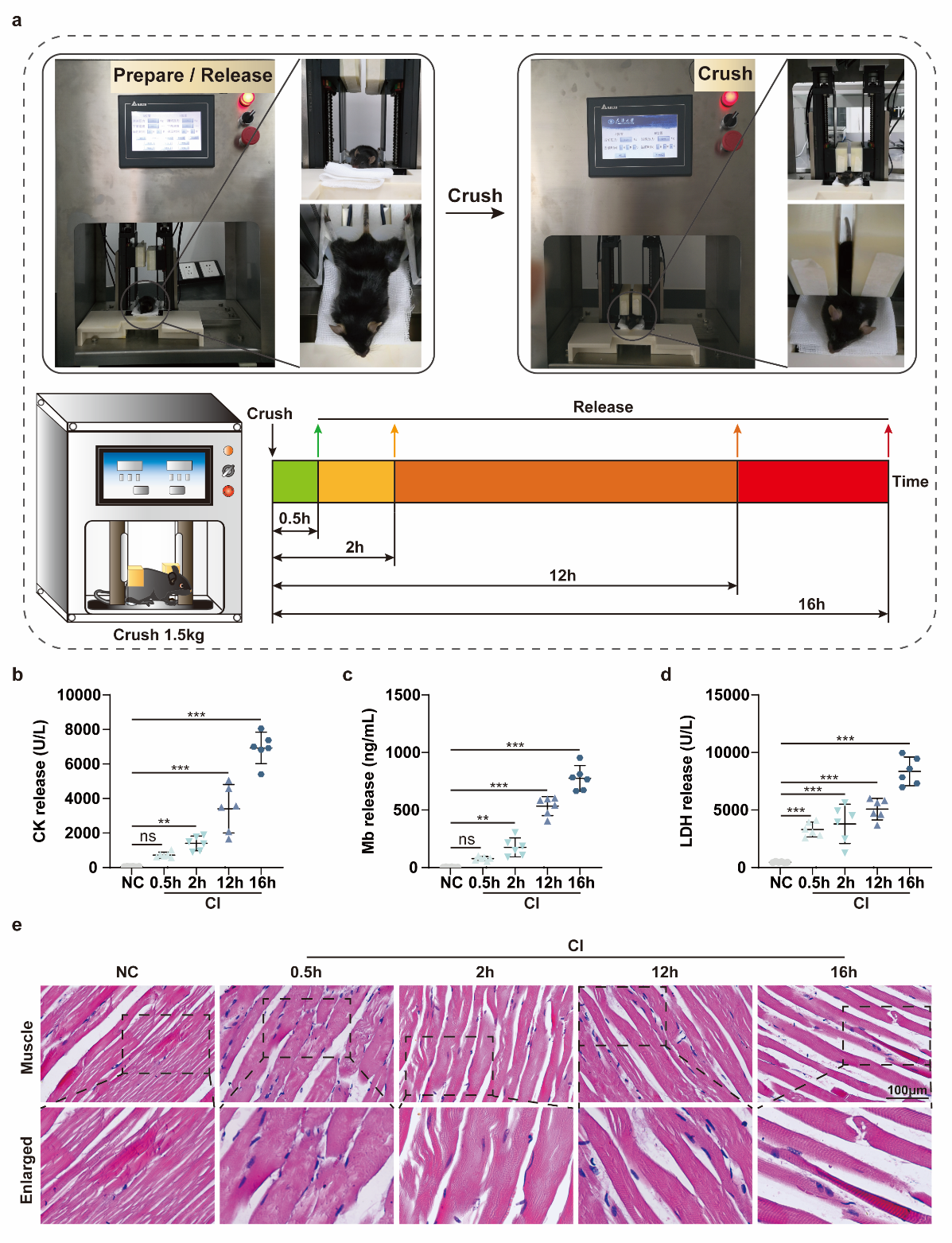


### Fig. S1 Successful establish the mouse CI model.

**a** Schematic diagram of establishing the mouse CI model. C57BL/6 J mice were crushed under 1.5 kg pressure for 0.5 h, 2 h, 12 h and 16 h, respectively. After the indicated times, directly decompress and immediately collect samples. **b-d** Biochemistry analyses the concentration of the CK, Mb and LDH in serum. **e** HE staining analyses skeletal muscle pathological changes under different crush time (0.5 h, 2 h, 12 h, 16 h) (original magnification: 400×; scale bar: 100 μm). One-way ANOVA was used in b-d (n = 6).


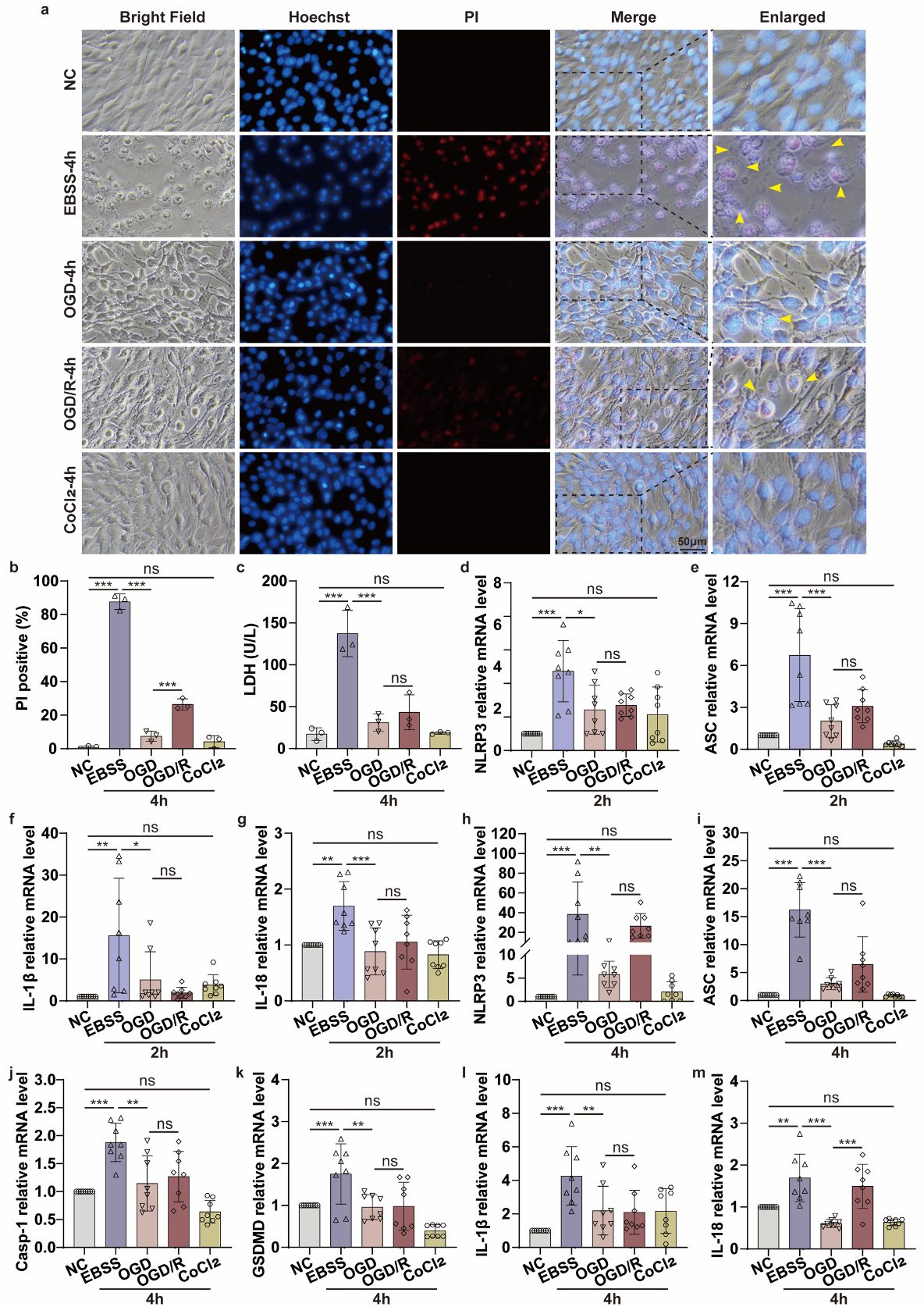


### Fig. S2 Ischemia and hypoxia treatment of C2C12 cells significantly attenuated the ischemic-induced pyroptosis via NLRP3/Casp-1/GSDMD pathway, Related to Fig. 3.

**a** Hoechst 33342/PI double staining to detect pyroptotic C2C12 cells after EBSS, OGD, OGD/R, CoCl2 treatment for 4 h. Bright-field microscopic images reveal the pyroptotic morphological manifestations of bubble-like formations, indicated by the yellow arrows (original magnification: 400×; scale bar: 50 μm). **b** PI-positive cell proportions. **c** Concentration of LDH in the supernatant. **d-g** qPCR analyses the mRNA level of pyroptosis associated molecular NLRP3, ASC, IL-1β and IL-18 in C2C12 cells after EBSS, OGD, OGD/R, CoCl2 treatment for 2 h. **h-m** qPCR analyses the mRNA level of pyroptosis associated molecular NLRP3, ASC, Casp-1, GSDMD, IL-1β and IL-18 in the C2C12 cells after EBSS, OGD, OGD/R, CoCl2 treatment for 4 h. One-way ANOVA was used in b-m (n = 8).


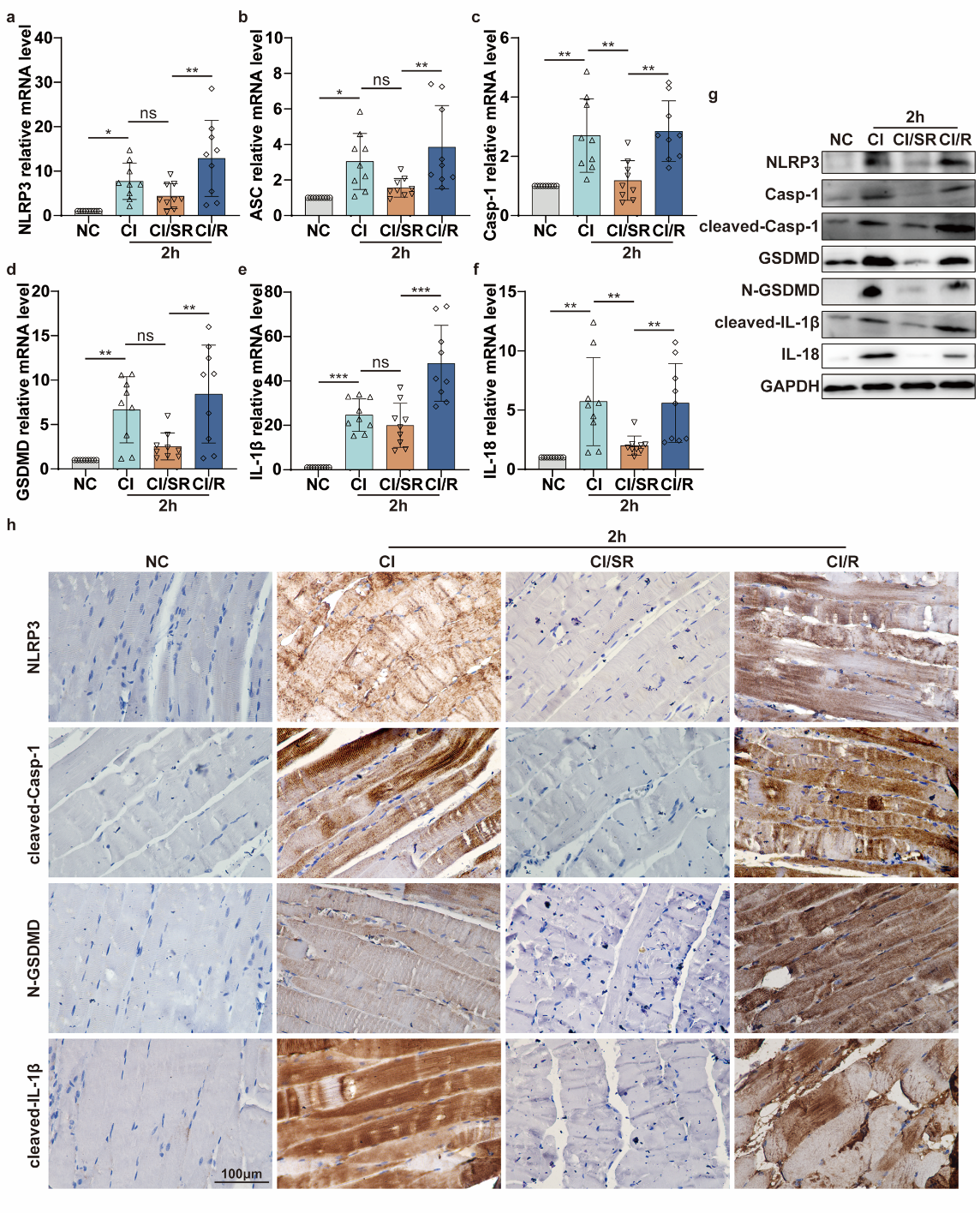


### Fig. S3 Delayed step-by-step release pressure significantly alleviates cell pyroptosis in skeletal muscle of crushed 2 h mediated by NLRP3/Casp-1/GSDMD pathway, Related to Fig. 6.

**a-f** qPCR analyses the mRNA level of pyroptosis associated molecular NLRP3, ASC, Casp-1, GSDMD, IL-1β, IL-18 in the skeletal muscle of mice crushed for 2 h followed by different decompression methods (NC, CI, CI/SR, CI/R). **g** WB analyses the protein expression level of NLRP3, Casp-1, cleaved-Casp-1, GSDMD, N-GSDMD, cleaved-IL-1β, IL-18 in the skeletal muscle of different groups. **h** IHC staining analyses the representative pyroptotic molecules NLRP3, cleaved-Casp-1, N-GSDMD, cleaved-IL-1β protein expression in skeletal muscle tissues of different groups (original magnification: 400×; scale bar: 100 μm). One-way ANOVA was used in a-f (n = 9).


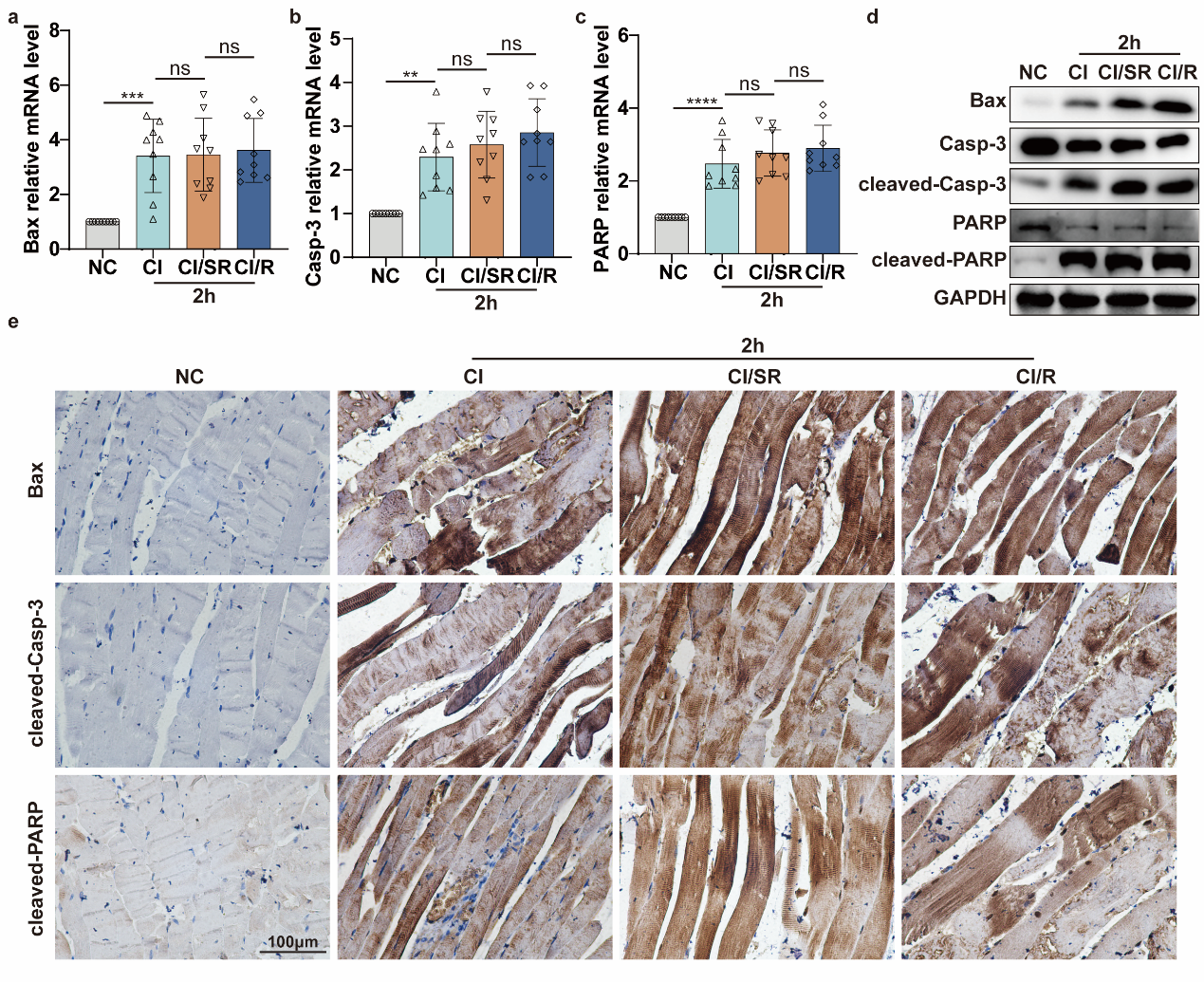


### Fig. S4 Delayed step-by-step release pressure not alter cell apoptosis in skeletal muscle of crushed 2 h mediated by Casp-3/PARP pathway, Related to Fig. 6.

**a-c** qPCR analyses the mRNA level of apoptosis associated molecular Bax, Casp-3, PARP in the skeletal muscle of mice crushed for 2 h followed by different decompression methods (NC, CI, CI/SR, CI/R). **d** WB analyses the protein expression level of Bax, Casp-3, cleaved-Casp-3, PARP and cleaved-PARP in the skeletal muscle of different groups. **e** IHC staining analyses the representative apoptotic molecules Bax, cleaved-Casp-3 and cleaved-PARP protein expression in muscle tissues of mice crushed for 2 h followed by different decompression methods (original magnification: 400×; scale bar: 100 μm). One-way ANOVA was used in a-c (n = 9).

**Supplementary Table**

| Gene names | Forward primer (5′-3′) | Reverse primer (5′-3′) |
| --- | --- | --- |
| NLRP3 | ATTACCCGCCCGAGAAAGG | TCGCAGCAAAGATCCACACAG |
| ASC | CTTGTCAGGGGATGAACTCAAAA | GCCATACGACTCCAGATAGTAGC |
| Casp-1 | ACAAGGCACGGGACCTATG | TCCCAGTCAGTCCTGGAAATG |
| GSDMD | CCATCGGCCTTTGAGAAAGTG | ACACATGAATAACGGGGTTTCC |
| IL-1β | TGGACCTTCCAGGATGAGGACA | GTTCATCTCGGAGCCTGTAGTG |
| IL-18 | GACTCTTGCGTCAACTTCAAGG | CAGGCTGTCTTTTGTCAACGA |
| Casp-8 | TGCTTGGACTACATCCCACAC | TGCAGTCTAGGAAGTTGACCA |
| BID | GCCGAGCACATCACAGACC | TGGCAATGTTGTGGATGATTTCT |
| Bax | TGAAGACAGGGGCCTTTTTG | AATTCGCCGGAGACACTCG |
| Casp-9 | TCCTGGTACATCGAGACCTTG | AAGTCCCTTTCGCAGAAACAG |
| Casp-3 | TGGTGATGAAGGGGTCATTTATG | TTCGGCTTTCCAGTCAGACTC |
| PARP | GGCAGCCTGATGTTGAGGT | GCGTACTCCGCTAAAAAGTCAC |
| GAPDH | AGGTCGGTGTGAACGGATTTG | TGTAGACCATGTAGTTGAGGTCA |

### Table S1. The primers used for qPCR detection.
