# Supplementary material for: Delayed step-by-step decompression with DSF alleviates skeletal muscle crush injury by inhibiting NLRP3/CASP-1/GSDMD pathway
Source: Cell Death Discov. 2023 Aug 1;9:280. doi: 10.1038/s41420-023-01570-3 (PMC10394048; doi:10.1038/s41420-023-01570-3)
Supplement: Supplementary file 2 — Original full length western blots [file 41420_2023_1570_MOESM2_ESM.docx]

**Supplementary material of original WB data for**

**Delayed step-by-step decompression with DSF alleviate skeletal muscle crush injury by inhibiting NLRP3/CASP-1/GSDMD pathway**

**The original WB image of Figure 1**


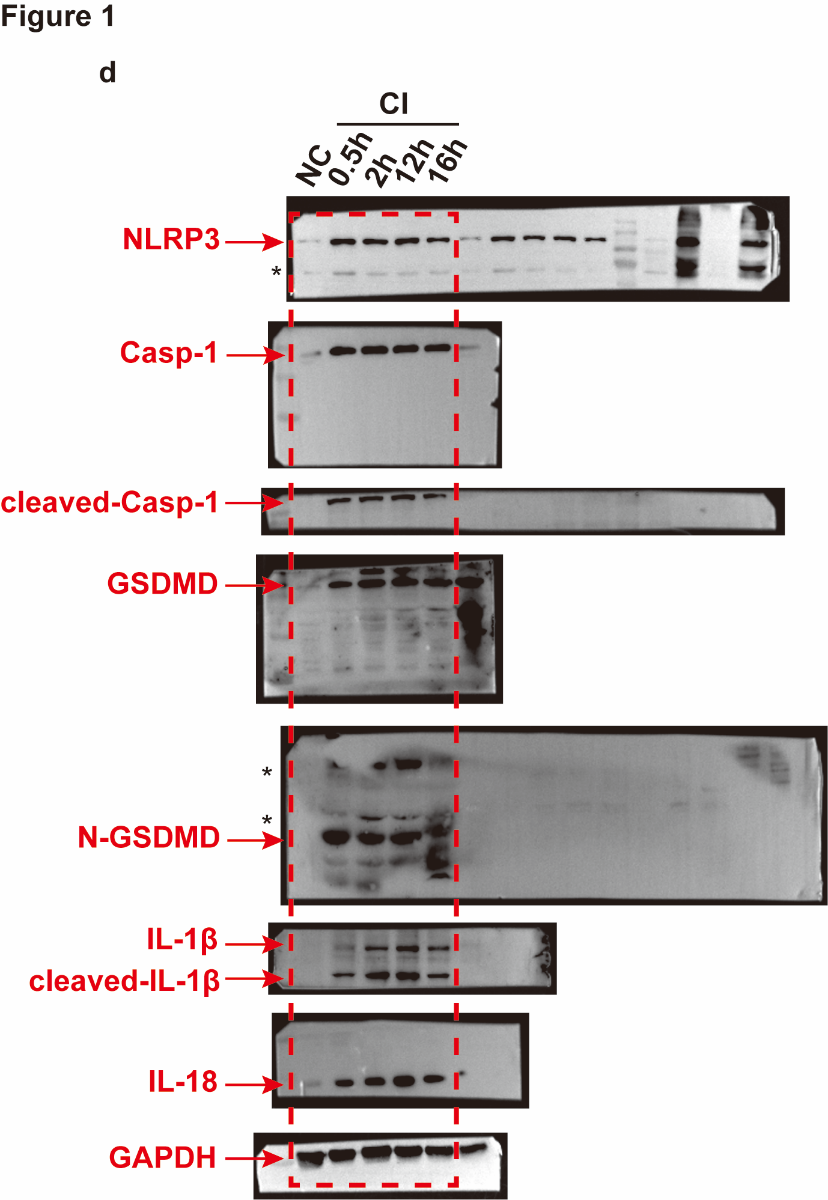


Notes：Gray asterisks denote a non-specific band.

**The original WB image of Figure 1**


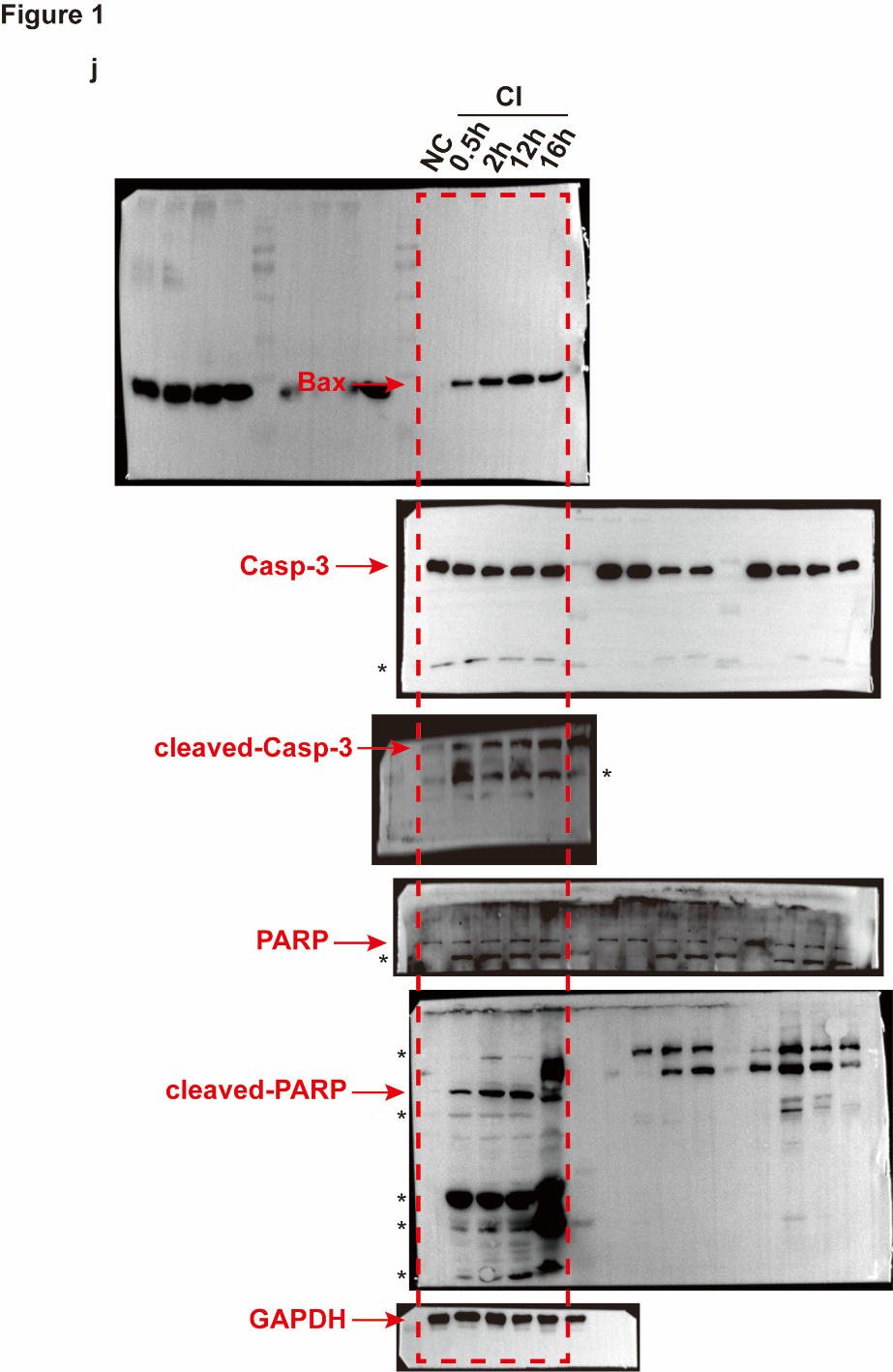


Notes：Gray asterisks denote a non-specific band.

**The original WB image of Figure 2**


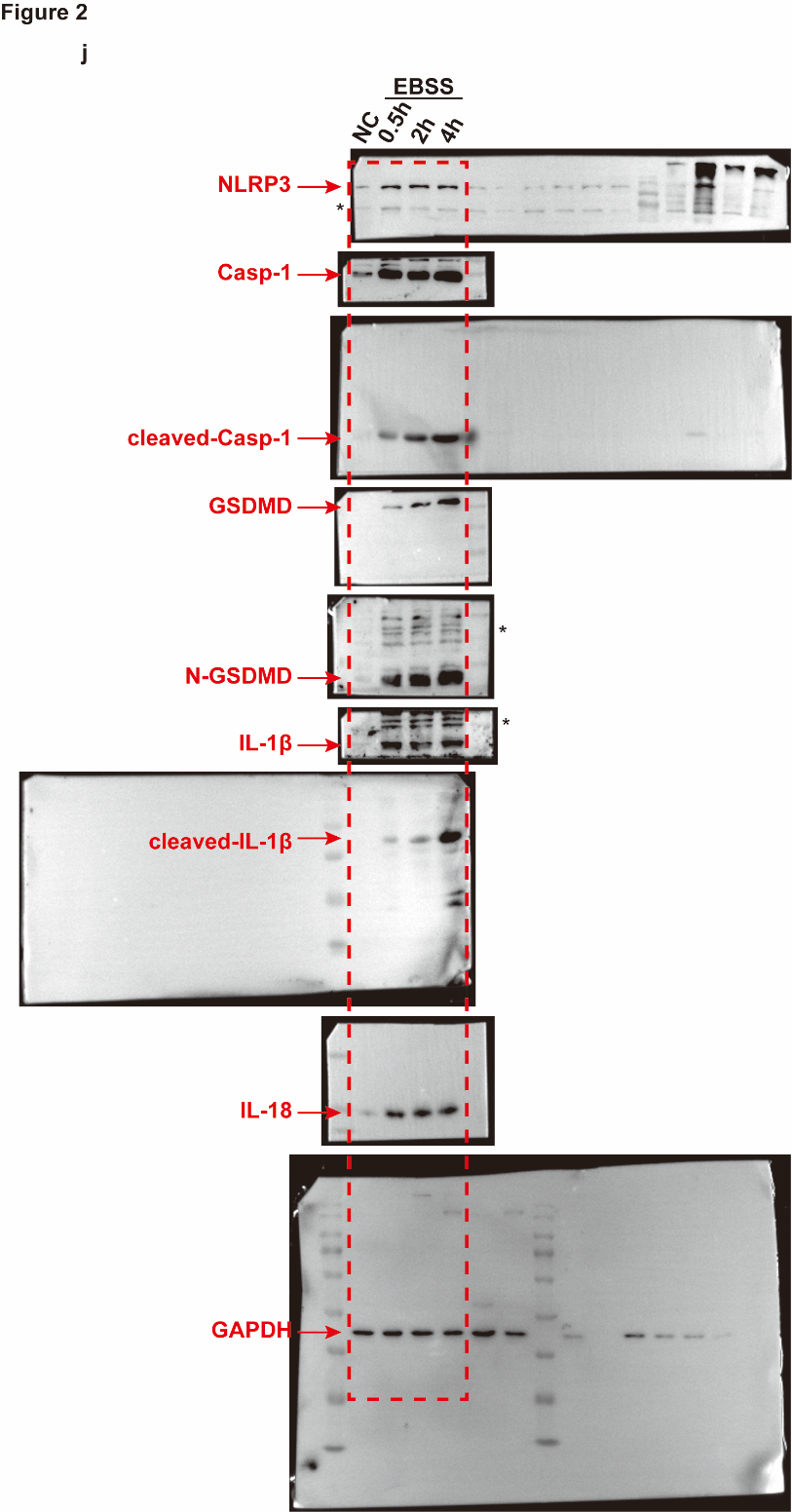


Notes：Gray asterisks denote a non-specific band.

**The original WB image of Figure 2**


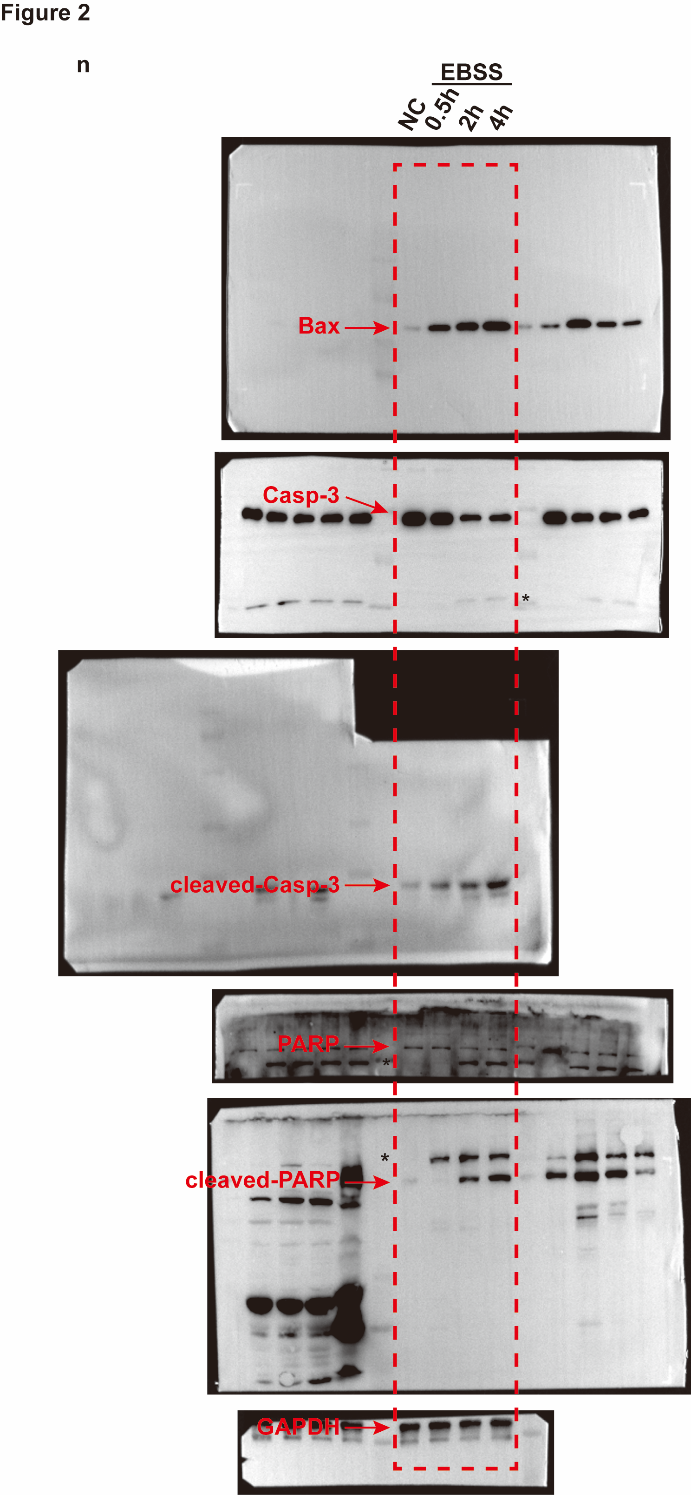


Notes：Gray asterisks denote a non-specific band.

**The original WB image of Figure 4**


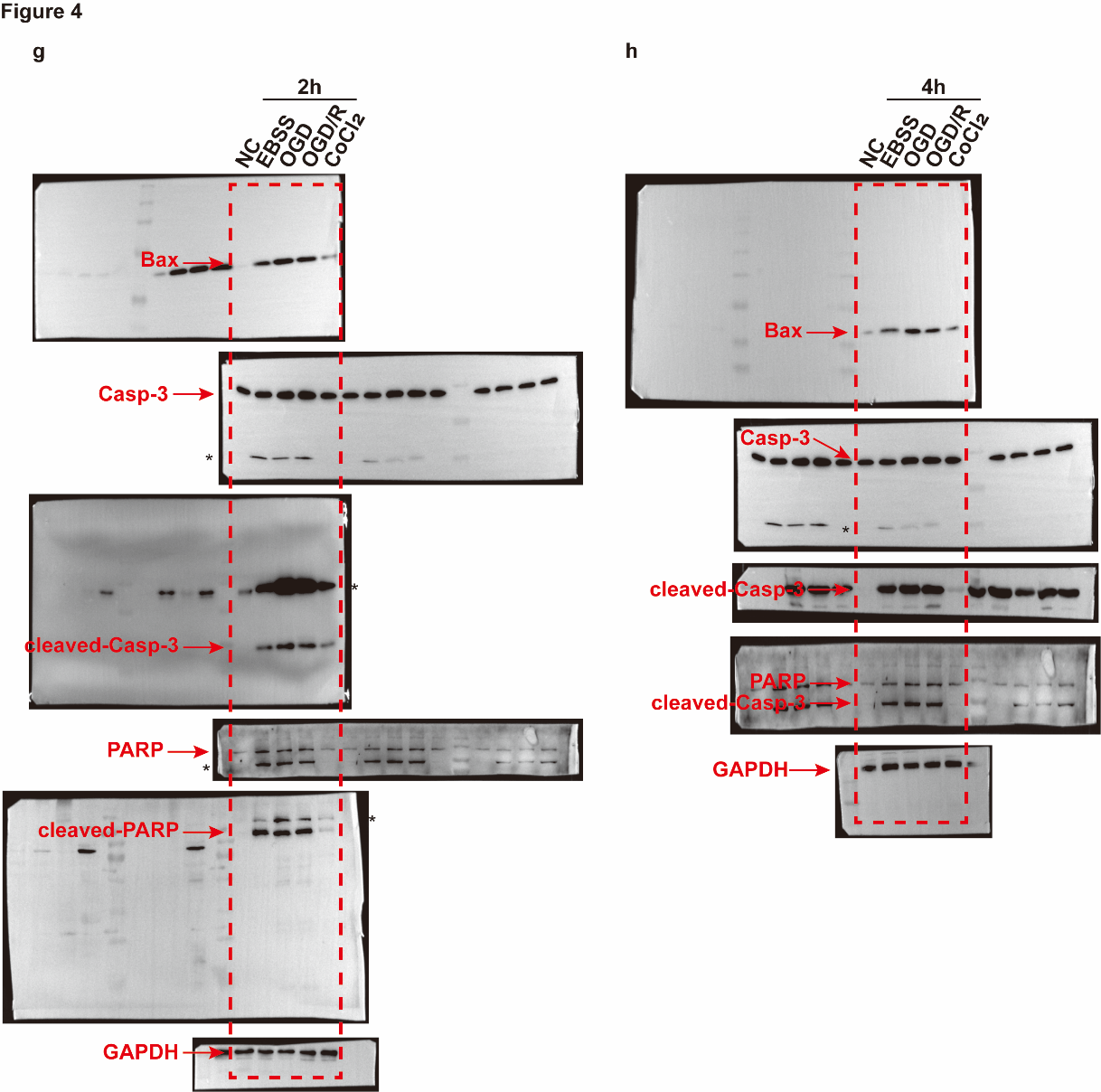


Notes：Gray asterisks denote a non-specific band.

**The original WB image of Figure 6**


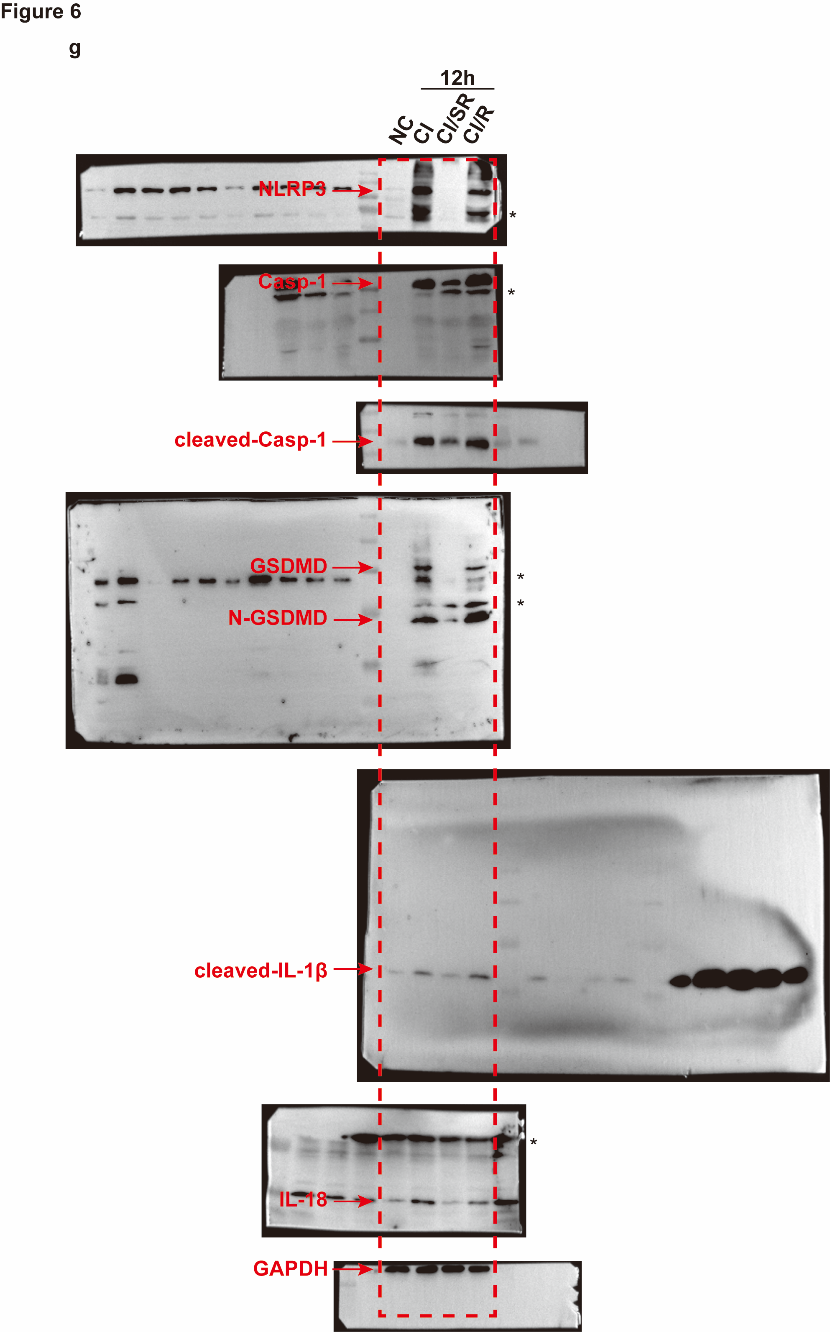


Notes：Gray asterisks denote a non-specific band.

**The original WB image of Figure 6**


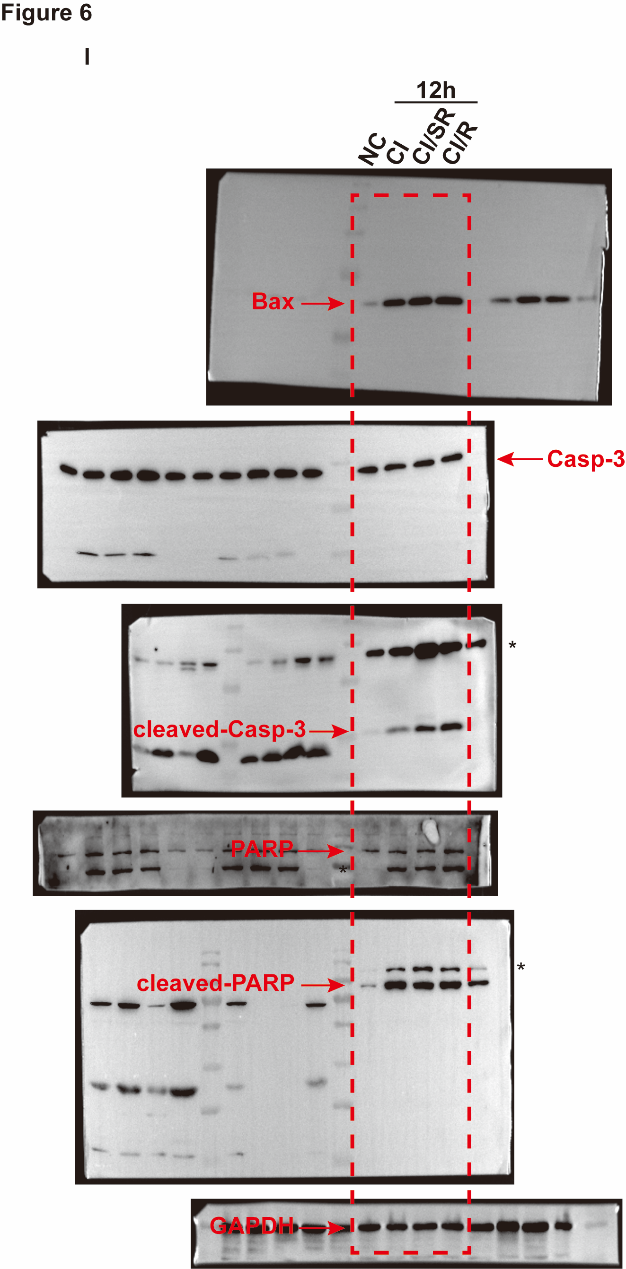


Notes：Gray asterisks denote a non-specific band.

**The original WB image of Figure 8**


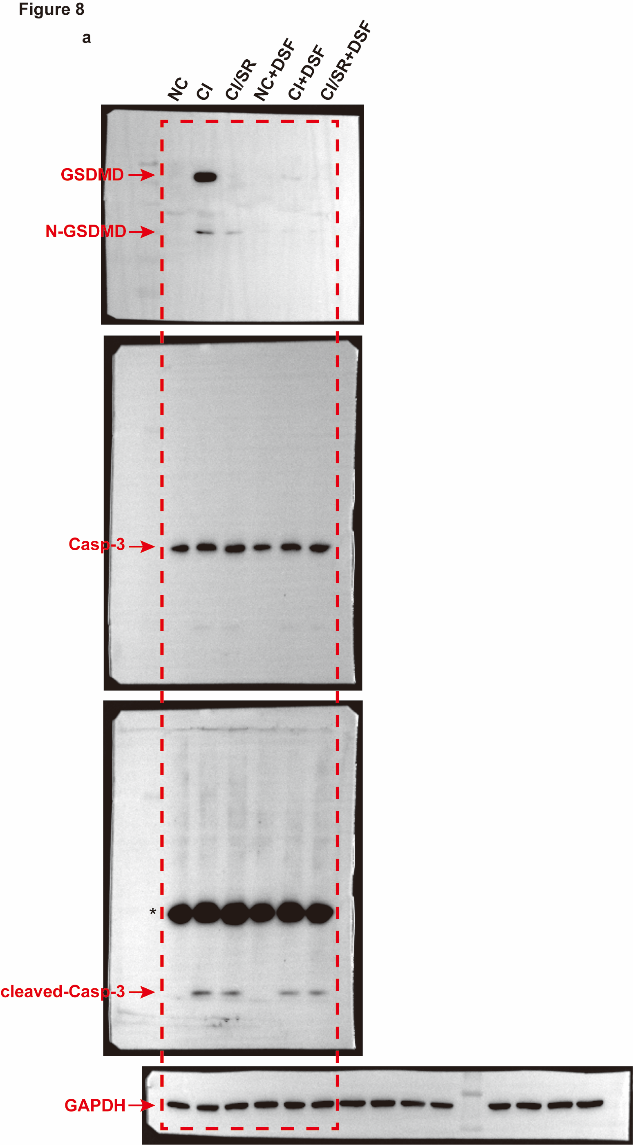


Notes：Gray asterisks denote a non-specific band.

**The original WB image of Figure S3**


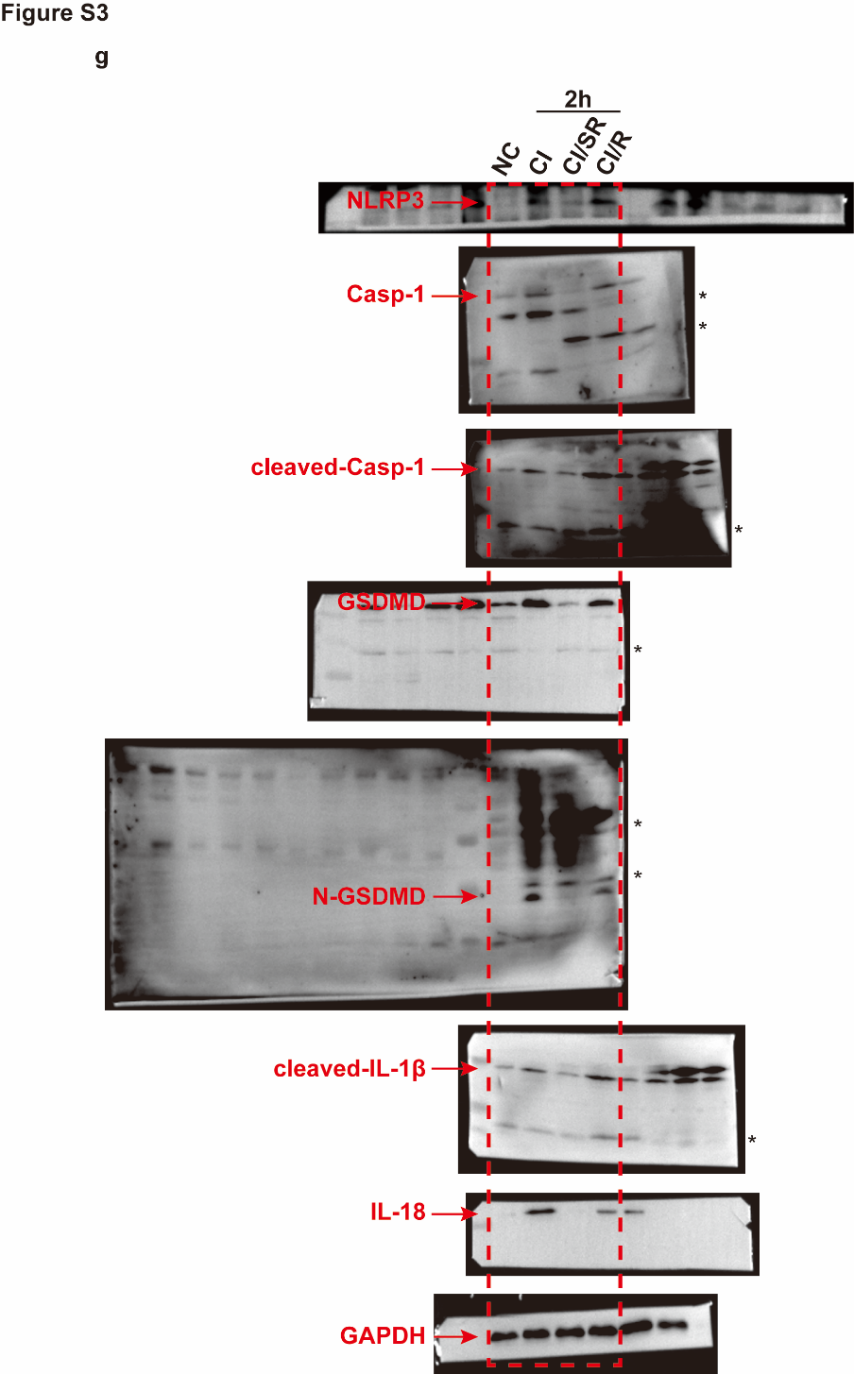


Notes：Gray asterisks denote a non-specific band.

**The original WB image of Figure S4**


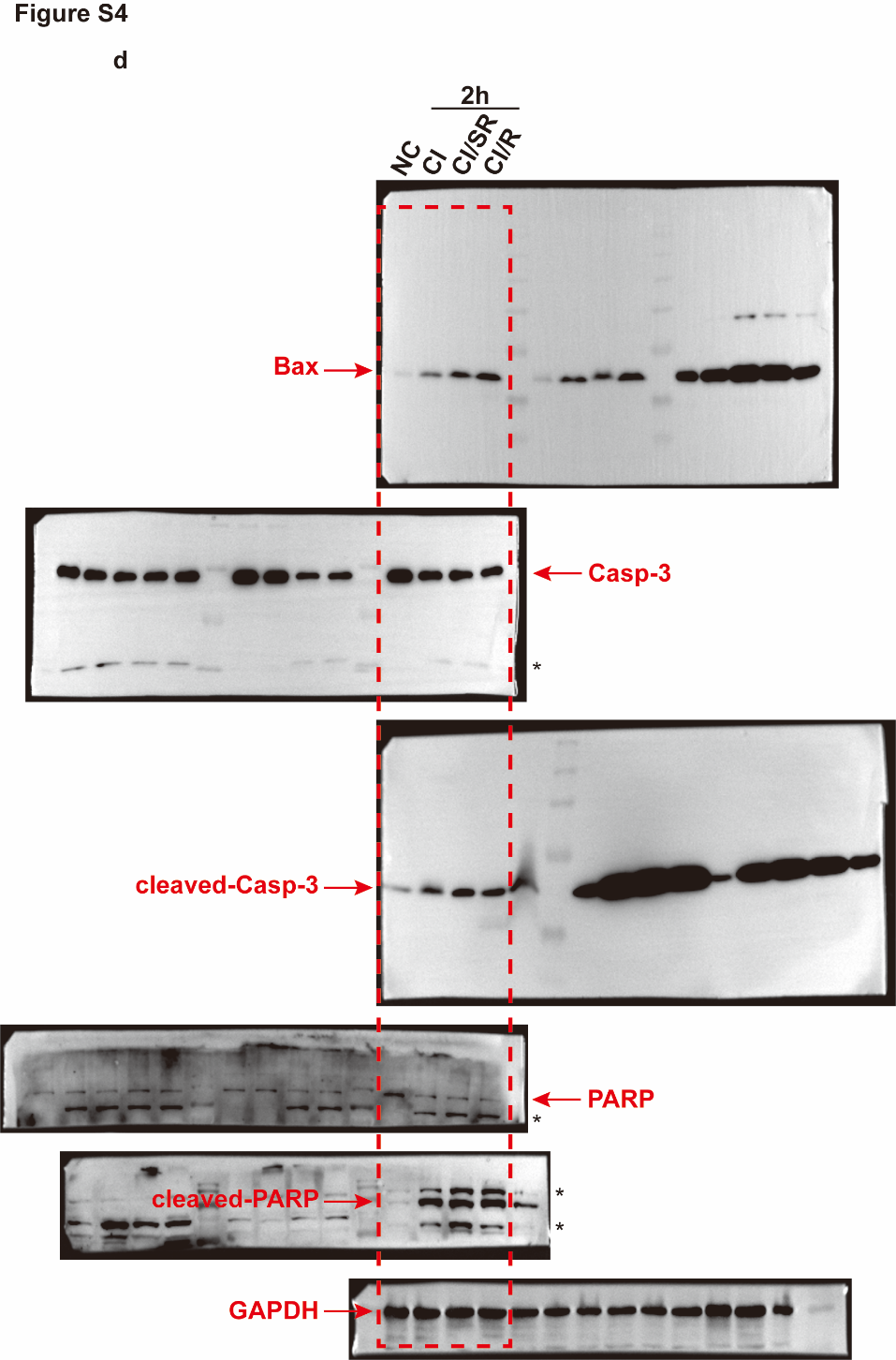


Notes：Gray asterisks denote a non-specific band.
